# Comparison of the Effectiveness of Low Carbohydrate Versus Low Fat Diets, in Type 2 Diabetes: Systematic Review and Meta-Analysis of Randomized Controlled Trials

**DOI:** 10.3390/nu14204391

**Published:** 2022-10-19

**Authors:** Tanefa A. Apekey, Maria J. Maynard, Monia Kittana, Setor K. Kunutsor

**Affiliations:** 1 School of Health, Leeds Beckett University, Leeds LS1 3HE, UK; 2 Diabetes Research Centre, University of Leicester, Leicester General Hospital, Gwendolen Road, Leicester LE5 4WP, UK; 3 Translational Health Sciences, Bristol Medical School, University of Bristol, Bristol BS8 1QU, UK

**Keywords:** low carbohydrate diet, low fat diet, type 2 diabetes, glucose, body weight, lipids, blood pressure, inflammation, adverse events, meta-analysis

## Abstract

The clinical benefit of low carbohydrate (LC) diets compared with low fat (LF) diets for people with type 2 diabetes (T2D) remains uncertain. We conducted a meta-analysis of randomized controlled trials (RCTs) to compare their efficacy and safety in people with T2D. RCTs comparing both diets in participants with T2D were identified from MEDLINE, Embase, Cochrane Library, and manual search of bibliographies. Mean differences and relative risks with 95% CIs were pooled for measures of glycaemia, cardiometabolic parameters, and adverse events using the following time points: short-term (3 months), intermediate term (6 and 12 months) and long-term (24 months). Twenty-two RCTs comprising 1391 mostly obese participants with T2D were included. At 3 months, a LC vs. LF diet significantly reduced HbA1c levels, mean difference (95% CI) of −0.41% (−0.62, −0.20). LC diet significantly reduced body weight, BMI, fasting insulin and triglycerides and increased total cholesterol and HDL-C levels at the short-to-intermediate term, with a decrease in the requirement for antiglycaemic medications at intermediate-to-long term. There were no significant differences in other parameters and adverse events. Except for reducing HbA1c levels and adiposity parameters at short-to-intermediate terms, a LC diet appears to be equally effective as a LF diet in terms of control of cardiometabolic markers and the risk of adverse events in obese patients with T2D.

## 1. Introduction

Type 2 diabetes (T2D) is a chronic and life-changing metabolic disorder that occurs when the pancreas does not produce enough insulin, or the body cannot effectively use the insulin it produces to regulate blood sugar. It is characterised by hyperglycaemia and associated with an unhealthy lifestyle [1,2,3]. Glycated hemoglobin also known as HbA1c is a preferred diagnostic test for T2D because it reflects an individual’s average blood glucose levels over the previous 3 months [4]. Reduction in levels of HbA1c is associated with reduction in T2D complications such as damage to the heart, nerves, blood vessels, eyes and kidneys, and death [4,5]. T2D is a major global public health concern due to the rising prevalence and its impact on the health of affected individuals, their families and the substantial costs associated with its management. According to the World Health Organisation, [3] the number of people with diabetes has quadrupled since 1980, with T2D as the vast majority (over 95%) of cases. In addition, the prevalence of T2D is now rising rapidly in both adults and children, and in low- and middle-income countries than in high-income countries. It is now the ninth leading cause of death, with over 6 million people dying from the disease in 2021 [6].

With appropriate interventions, early detection and support, T2D can be prevented, delayed, managed and even result in remission [7,8]. Modifiable risk factors such as active lifestyles, maintaining a healthy body mass index (BMI), smoking cessation, reduced alcohol intake and a healthy diet have been shown to be effective at preventing and delaying the onset of T2D as well as its remission [9,10,11]. For example, high resistant starch rice has commonly been used as an effective food product to prevent diabetes via its ability to control gluconeogenesis, promote glycogenesis, maintain glucose and lipid homeostasis, and improve pancreatic function [12]. Another nutritional food product known to combat chronic diseases including diabetes is barley [13]. However, there are variations in nutritional advice by guideline recommendations and healthcare systems [8,14,15] and in the effectiveness of dietary interventions [1,16,17] for people living with T2D. In addition, fat and carbohydrate recommendations for adults with T2D vary across diabetes organisations [18]. The most common variations in dietary approaches to the treatment of T2D are in the amount and type of carbohydrate and fat consumed. However, it is not clear as to whether low carbohydrate (LC) or low fat (LF) diet is superior for weight loss and the treatment of T2D. Similarly, there is no agreed definition for LC diets, hence there is variation across studies [18], ranging from 20 g/day (<10% total energy) to 130 g/day (<26% total energy) carbohydrates [19]. For the purposes of this review, LC diet was defined as diets with less than 130 g/day or less than 26% of total energy from carbohydrates (based on an energy intake of 2000 kcal/day). This definition of LC (26% total energy) was based on the proposed classifications of dietary carbohydrate intake by Feinman et al. [18], and to avoid overlap of carbohydrate intake between the intervention and comparator groups. A recent systematic review evaluated the effectiveness of low and very LC diets on T2D remission but only 78% of the comparator diets were LF [20]. The UK Scientific Advisory Committee on Nutrition (SACN) [18] also reviewed the evidence on LC diets compared to current UK government advice on carbohydrate intake for adults with type 2 diabetes. However, LC diet in this review was defined as carbohydrate intake ranging from 14% to 50% total energy per day. Both reviews did not consider other important outcomes such as liver markers, renal function, and systolic and diastolic blood pressure (SBP and DBP). We conducted a meta-analysis of randomized controlled trials (RCTs) to compare the efficacy and safety of LC diets compared with LF diets for people with T2D using a comprehensive list of cardiometabolic outcomes including blood pressure and markers of renal and liver function.

## 2. Materials and Methods

### 2.1. Data Sources and Search Strategy

This review was conducted using a predefined protocol, registered in the PROSPERO prospective register of systematic reviews (CRD42021254388), and in accordance with PRISMA and MOOSE guidelines [21,22] (Tables S1 and S2). A systematic search for RCTs was carried out on PubMed, MEDLINE, Embase, Web of Science, Clinical Trials.gov, and the Cochrane electronic databases from January 1981 till July 2021. The search was updated on 12 October 2022 following initial review. The computer-based searches combined terms related to the exposures (e.g., low carbohydrate diet, low fat diet, calorie-restricted diet) and population (e.g., prediabetes, type 2 diabetes) in humans, without any language restriction. Full details of the MEDLINE search strategy are provided in Table S3. Two authors (TA and SKK) independently screened titles and abstracts of the retrieved citations to assess their suitability for potential inclusion, which was followed by the acquisition of full texts for detailed evaluation. Full-text evaluation was also independently conducted by two authors (TA and SKK). Disagreements regarding eligibility of an article were discussed and resolved by consensus. The reference lists of relevant studies and review articles were manually scanned for additional studies.

### 2.2. Eligibility Criteria

Studies were eligible if they were randomized controlled, open or blinded trials that: (i) enrolled adults classified as prediabetes and those living with T2D regardless of medication use, glucose and glycated haemoglobin (HbA1c) levels, and comorbidities; (ii) compared a LC diet (<26% of total energy or <130 g of carbohydrate a day) with a LF diet (>26% of total energy or >130 g of carbohydrates a day); (iii) reported at least 12 weeks duration of the trial; (iv) and reported on any of the outcomes below.

### 2.3. Outcomes Evaluated

Our primary outcomes of interest were measures of glycaemia including fasting plasma glucose, mean glucose, and HbA1c levels. Secondary outcomes were measures of body composition (e.g., body weight, BMI, waist circumference, total fat free mass); cardiovascular risk markers (e.g., SBP, DBP, lipids, fasting insulin, measures of insulin resistance, measures of inflammation such as C-reactive protein (CRP)); liver function tests (e.g., alanine aminotransferase (ALT), aspartate aminotransferase (AST), gamma glutamyltransferase (GGT)); renal function tests (e.g., creatinine, estimated glomerular filtration rate (GFR), urinary albumin); measures of medication changes (e.g., medication effect score (MES)); and adverse events. Non-randomized studies that compared the desired interventions were excluded.

### 2.4. Data Extraction and Risk of Bias Assessment

Two authors (TA and SKK) independently extracted data, with inconsistencies resolved by discussion. A predesigned data extraction form was used to extract all the relevant information on publication date, geographical location, study design characteristics (e.g., randomization, allocation concealment, blinding, duration), population (e.g., baseline age, percentage of males, baseline BMI and HbA1c), intervention and comparator, and outcomes. Outcome data were extracted for the specific time points reported by the trials. For multiple publications of studies using data from the same trial, non-overlapping data based on the most comprehensive results were extracted. We used the Cochrane Collaboration’s risk of bias tool to assess the risk of bias of included RCTs [23]. This tool evaluates seven possible sources of bias: random sequence generation, allocation concealment, blinding of participants and personnel, blinding of outcome assessment, incomplete outcome data, selective reporting and other bias. For each individual domain, studies were classified into low, unclear and high risk of bias. We also used the Grading of Recommendations Assessment, Development and Evaluation (GRADE) tool to assess the quality of the body of evidence, based on study limitations, inconsistency of effect, imprecision, indirectness and publication bias [24]. 

### 2.5. Statistical Analysis

Summary measures of effect were presented as relative risks (RRs) (95% CIs) for binary outcomes and mean differences (95% CIs) for continuous outcomes and. Relative risks and 95% CIs were estimated from the extracted raw counts for the interventions and comparators. For studies that reported data such as medians (ranges and 95% CIs) and means (SDs and standard errors), these were converted to means and standard deviations using methods described by Hozo and colleagues [25]. For each outcome, effect estimates (RRs and mean differences) were estimated for the time points of 3 months (±1 month), 6 months (+2 months), 12 months (±3 months), and 24 months (±6 months), based on the distribution of the time points reported by the eligible studies and to maintain some consistency with that of a previous review [20]. The time points were categorised as short-term (3 months), intermediate term (6 and 12 months) and long-term (24 months). Measures of effect were pooled using random effects models to minimize the effect of heterogeneity [26]. Where appropriate, fixed effects models were used in parallel analyses. We planned to investigate sources of heterogeneity using subgroup analysis and random effects meta-regression [27] as well as assess for small study effects using formal tests such as Begg’s funnel plots [28] and Egger’s regression symmetry test [29]. However, these could not be performed because of the limited number of studies (<10) for each outcome assessed. All analyses were conducted using Stata version MP 16 (Stata Corp, College Station, TX, USA). For outcomes that could not be pooled, a narrative synthesis was used to summarise the results. 

## 3. Results

### 3.1. Study Identification and Selection

**Figure 1:**
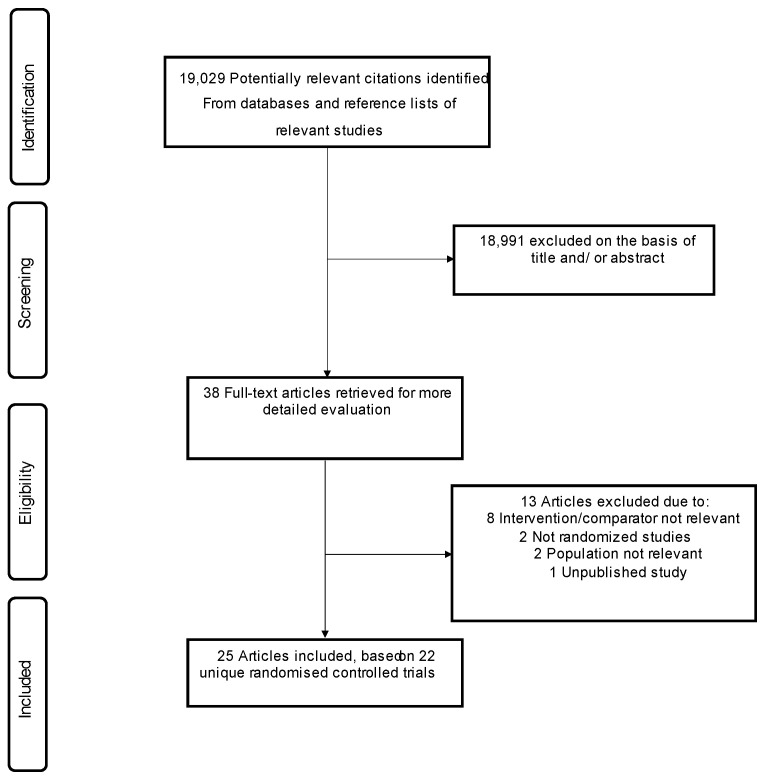
PRISMA flow diagram.

Our initial search of relevant databases and manual scanning of reference lists identified 19,029 potentially relevant citations. After screening based on titles and abstracts, 38 articles remained for full text evaluation. Following detailed assessments, 13 articles were excluded because (i) intervention/comparator was not relevant (n = 8); (ii) they were not randomized studies (n = 2); (iii) population was not relevant (n = 2); and (iv) was based on a conference presentation (n = 1). The remaining 25 articles [30,31,32,33,34,35,36,37,38,39,40,41,42,43,44,45,46,47,48,49,50,51,52,53,54] which were based on 22 unique RCTs, met our inclusion criteria and were included in the meta-analysis (Figure 1). 

### 3.2. Study Characteristics and Risk of Bias

Table 1 and Table 2 summarizes the key characteristics of the RCTs included in the review. In aggregate, the included trials published between 2003 and 2022, comprised 1391 participants (711 assigned to LC diet and 680 assigned to LF diet). All RCTs were open-labelled and recruited patients with T2D, with the majority being obese and/or overweight. No study was identified to have recruited people with prediabetes. The mean baseline age, BMI, HbA1c, and duration of T2D of participants ranged from 36.8–67.0 years, 25.8–38.1 kg/m^2^, 6.0–9.1 %, 0.3–13.5 years, respectively; with weighted means of 57.2 years, 34.4 kg/m^2^, 7.8%, and 7.6 years, respectively. Overall, 8 studies were conducted in Asia (China, Iran, Israel, Japan, and Taiwan), 6 in North America (USA) and 5 in Europe (Denmark, Italy, Sweden, and UK) and 3 in Australasia (Australia). The mean duration of trials or interventions in the trials ranged from 2–24 months with a weighted mean of 12.4 months. Not all studies provided information on dietary adherence. Exercise was usually encouraged as part of the dietary interventions in some studies but was not evaluated in separate analyses. Using the Cochrane Risk of Bias tool, all 22 trials demonstrated a high risk of bias in blinding of participants & personnel; all but 2 demonstrated a high risk of bias in blinding of outcome assessments; and 7 trials demonstrated a high risk of bias in 3 or more domains (Figure S1). 

### 3.3. Measures of Glycaemia

**Figure 2:**
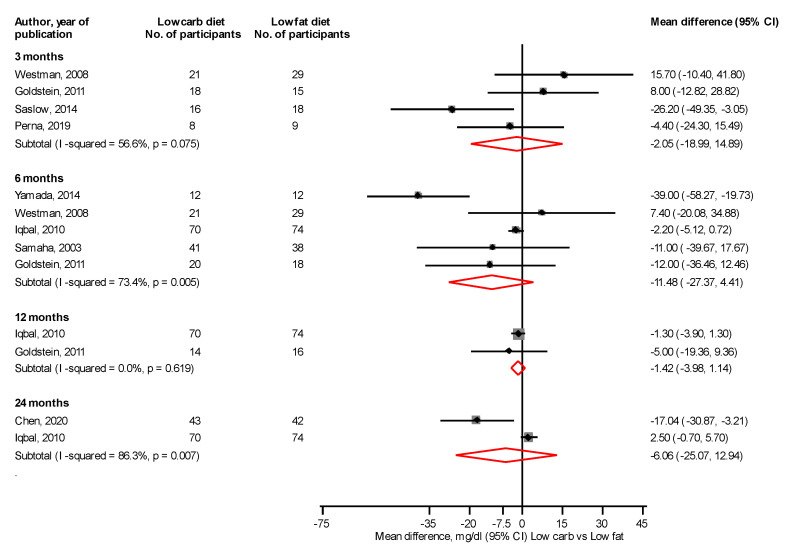
Low carbohydrate versus low fat diet and fasting glucose levels [31,34,36,41,42,43,50,51].

**Figure 3:**
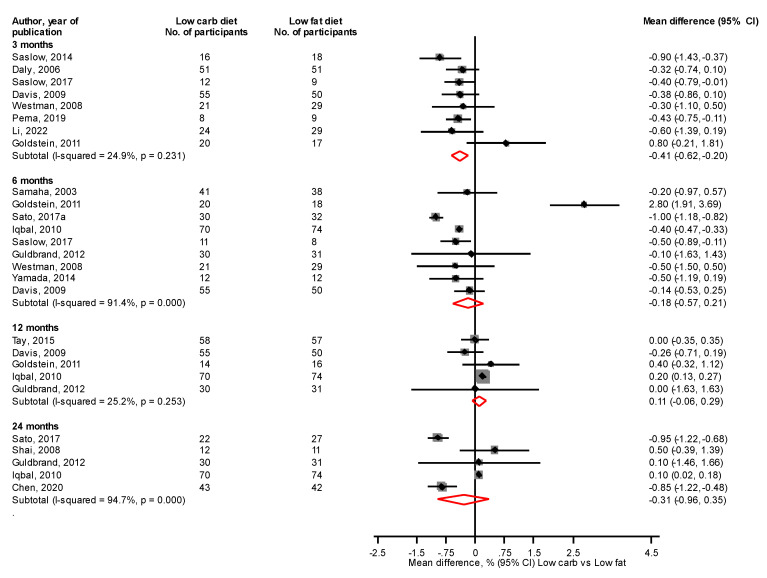
Low carbohydrate versus low fat diet and HbA1c levels [31,32,33,34,35,36,41,42,42,44,46,47,50,51,52,54].

Comparing LC with LF diets, there were no significant differences in fasting glucose levels at 3, 6, 12, and 24 months: mean differences (95% CIs) of −2.05 (−18.99, 14.89), −11.48 (−27.37, 4.41), −1.42 (−3.98, 1.14) and −6.06 (−25.07, 12.94) mg/dL, respectively (Figure 2). At 3 months, a LC vs. LF diet significantly reduced HbA1c levels, mean difference (95% CI) of −0.41% (−0.62, −0.20), with no differences at 6, 12, and 24 months: mean differences (95% CIs) of −0.18% (−0.57, 0.21), 0.11% (−0.06, 0.29), and −0.31% (−0.96, 0.35) mg/dL, respectively (Figure 3).

### 3.4. Body Composition

**Figure 4:**
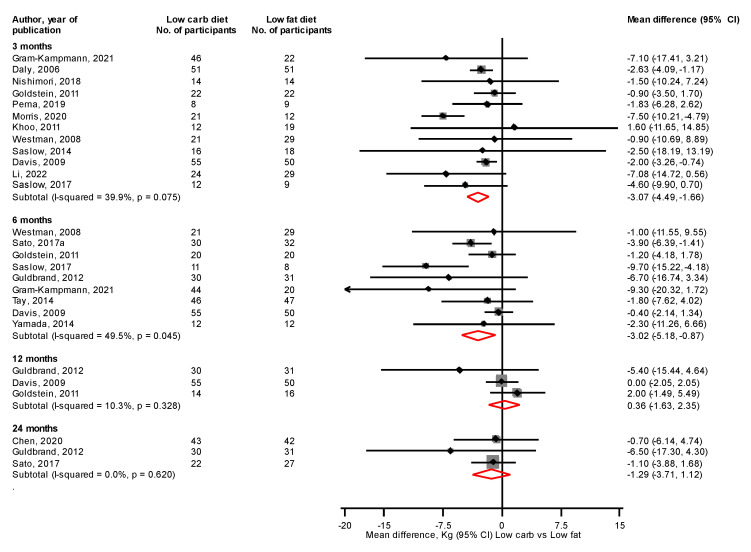
Low carbohydrate versus low fat diet and body weight [31,32,33,34,35,38,39,40,41,43,44,45,46,48,50,51,52,53].

**Figure 5:**
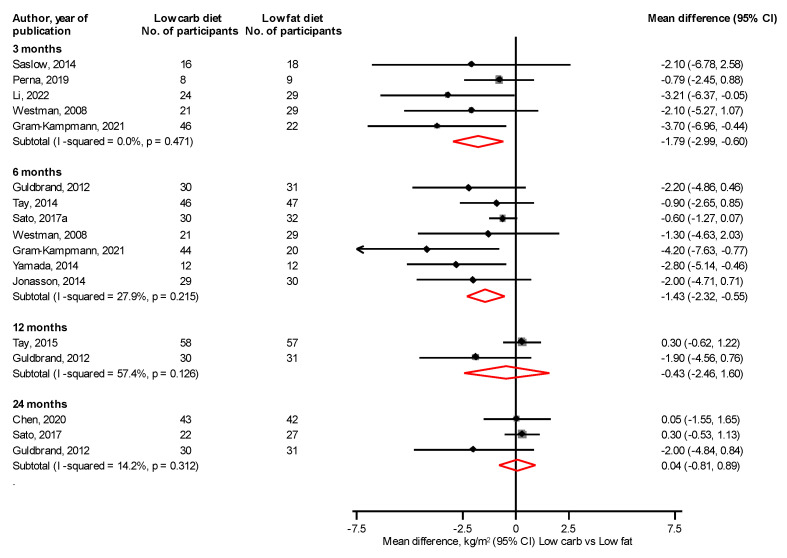
Low carbohydrate versus low fat diet and body mass index [31,35,37,41,43,45,46,48,50,51,52,53,54].

**Figure 6:**
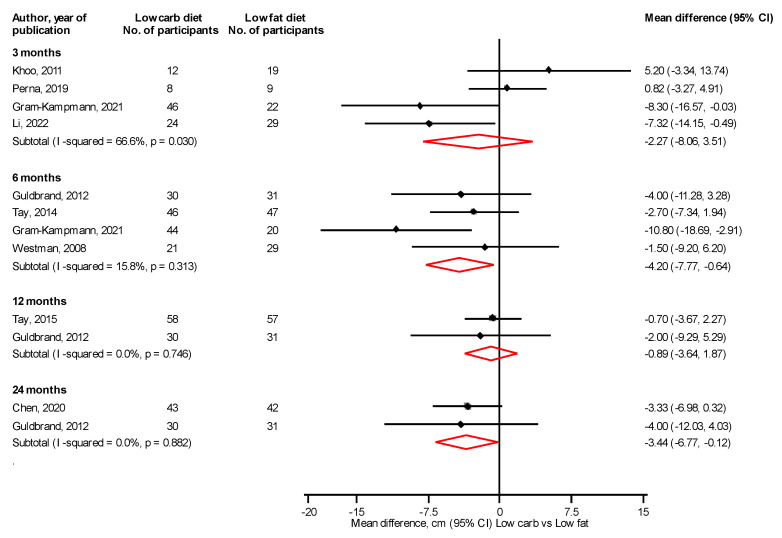
Low carbohydrate versus low fat diet and waist circumference [31,35,38,41,48,50,52,53,54].

At 3 and 6 months, a LC vs. LF diet significantly reduced body weight, mean differences (95% CIs) of −3.07 kg (−4.49, −1.66) and −3.02 kg (−5.18, −0.87), respectively; with no differences at 12 and 24 months: mean differences (95% CIs) of 0.36 kg (−1.63, 2.35) and −1.29 kg (−3.71, 1.12), respectively (Figure 4). At 3 and 6 months, a LC vs. LF diet significantly reduced BMI, mean differences (95% CIs) of −1.79 kg/m^2^ (−2.99, −0.60) and −1.43 kg/m^2^ (−2.32, −0.55), respectively, with no differences at 12 and 24 months: mean differences (95% CIs) of −0.43 kg/m^2^ (−2.46, 1.60) and 0.04 kg/m^2^ (−0.81, 0.89), respectively (Figure 5). At 6 and 24 months, a LC vs. LF diet significantly reduced waist circumference: mean differences (95% CIs) of −4.20 cm (−7.77, −0.64) and −3.44 cm (−6.77, −0.12), respectively, with no differences at 3 and 12 months: mean differences (95% CIs) of −2.27 cm (−8.06, 3.51) and −0.89 cm (−3.64, 1.87), respectively (Figure 6). Results from single reports showed no significant differences in fat free mass at 3, 6, and 12 months comparing a LC with a LF diet (Figure S2).

### 3.5. Blood Pressure

**Figure 7:**
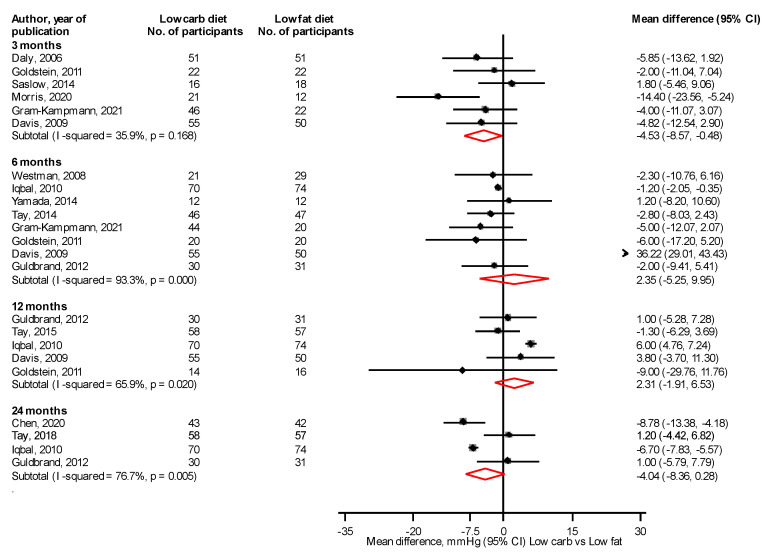
Low carbohydrate versus low fat diet and systolic blood pressure [31,32,33,34,35,36,39,43,48,49,50,51,53,54].

When LC and LF diets were compared, except for a reduction in SBP at 3 months: mean difference (95% CI) of −4.53 mmHg (−8.57, −0.48) (Figure 7), there were no significant differences in SBP and DBP at all other time periods (Figure 7; Figure S3).

### 3.6. Lipids

A comparison of LC and LF diets showed there were significant increases in total cholesterol levels at 6 and 12 months: mean differences (95% CIs) of 2.24 mg/dL (0.50, 3.99) and 6.41 mg/dL (3.10, 9.73), respectively, with no significant differences at 3 and 24 months (Figure S4). There were no significant differences in low-density lipoprotein cholesterol (LDL-C) levels at 3, 6, 12, and 24 months (Figure S5). The LC diet significantly increased high-density lipoprotein cholesterol (HDL-C) levels compared with the LF diet at 3 and 12 months: mean differences (95% CIs) of 2.85 mg/dL (0.33, 5.36) and 1.62 mg/dL (1.20, 2.04), respectively, with no significant differences at 6 and 24 months (Figure S6). At 3 and 6 months, a LC vs. LF diet significantly reduced levels of triglycerides, mean differences (95% CIs) of −25.33 mg/dL (−44.78, −5.87) and −20.62 mg/dL (−37.91, −3.32), respectively, with no differences at 12 and 24 months (Figure S7). A LC diet reduced the total cholesterol/HDL-C at 3 months: mean difference (95% CI) of −0.33 (−0.56, −0.11) (Figure S8). 

### 3.7. Measures of Inflammation

There were no significant differences in CRP levels between the LC and LF diets at 3, 6, 12, and 24 months (Figure S9). Results from single reports showed that a LC diet reduced interleukin-6 (IL-6) levels at 6 months, with no significant difference at 3 months (Figure S10). 

### 3.8. Other Cardiovascular Risk Markers

The LC diet significantly reduced fasting insulin levels compared with the LF diet at 3 months: mean difference (95% CI) of −2.83 µIU/mL (−4.73, −0.93), but with no significant differences at 6, 12, and 24 months (Figure S11). Results from single reports showed no significant differences in HOMA2-IR at all time points comparing a LC with a LF diet (Figure S12). The LC diet significantly reduced HOMA-IR compared with the LF diet at 3 months: mean difference (95% CI) of −0.71 (−1.05, −0.37) (Figure S13). A single report showed no significant difference in HOMA2-%B at all time points comparing a LC with a LF diet (Figure S14).

### 3.9. Liver Function

At 3 months, a LC vs. LF diet significantly reduced ALT levels, mean difference (95% CI) of −8.86 U/L (−17.09, −0.62). Results from a single report showed that at 6 months, a LC vs. LF diet reduced ALT levels (Figure S15). Comparing a LC with a LF diet, there were no significant differences in AST and GGT levels at 3 and 6 months (Figures S16 and S17).

### 3.10. Renal Function

Based on a single report, a LC vs. LF diet significantly increased creatinine levels at 6 months; with no differences at 3 and 24 months (Figure S18). Results from single reports showed no significant differences in estimated GFR at 3, 6, and 24 months comparing a LC with a LF diet (Figure S19). Results from single reports showed no significant differences in microalbumin at 3, 6, and 12 months comparing a LC with a LF diet (Figure S20). Results from single reports showed a significant increase in urea at 3 months, with no significant differences at 6 and 12 months comparing a LC with a LF diet (Figure S21). Results from single reports showed no significant differences in urinary albumin at 6 and 24 months comparing a LC with a LF diet (Figure S22). Results from single reports showed a significant increase in uric acid at 3 months with no difference at 24 months comparing a LC with a LF diet (Figure S23).

### 3.11. Medication Changes

The LC diet achieved a significant reduction in antiglycemic MES compared with the LF diet at 12 and 24 months, with no significant difference at 6 months (Figure S24).

### 3.12. Adverse Events

With respect to the two diets, there was no significant difference in risk of adverse events (e.g., musculoskeletal ailments with exercise training, hypoglycaemia and gastrointestinal complaints): RR (95% CI) of 1.27 (0.74–2.18; *p* = 0.38) (Figure S25).

### 3.13. GRADE Summary of Findings

The GRADE working group recommends up to 7 patient-important outcomes to be listed in the “summary of findings” tables in systematic reviews [24]. In addition to the primary outcomes of fasting glucose and HbA1c levels, we selected body weight, BMI, SBP, and total cholesterol based on their frequency of reporting. We also included the outcome of adverse events since it is recommended that the 7 selected outcomes should include a safety outcome. GRADE ratings for the outcomes are reported in Table S4. GRADE quality of the evidence ranged from moderate to very low.

## 4. Discussion

### 4.1. Key Findings

Given the persisting uncertainty regarding the net clinical benefits of LC diets compared with LF diets for people with T2D remains uncertain, we conducted an aggregate meta-analysis to compare the efficacy and safety of LC with LF diets in people with T2D. For the primary endpoints, LC compared with LF diet reduced HbA1c levels only at the 3-month time point, with no differences in fasting glucose levels at all time points. For body composition measures, LC diet reduced body weight, BMI, and waist circumference mostly at short and intermediate terms. LC diet reduced SBP at 3 months, with no significant differences in SBP and DBP at other time points. Findings for lipid parameters were inconsistent: at short and intermediate terms, there were reductions in levels of triglycerides and total cholesterol/HDL-C ratio; increases in total cholesterol and HDL-C; and no significant differences in levels of LDL-C. There were no significant differences for CRP, AST, GGT, and the risk of adverse events comparing LC with LF diet. LC diet significantly reduced fasting insulin and ALT at short-to-intermediate terms and antiglycemic MES at intermediate-to-long terms. Results from single reports showed LC diet reduced IL-6 levels; increased levels of creatinine and urea; with no differences for fat free mass, HOMA2-IR, HOMA2-%B, estimated GFR, microalbumin, and urinary albumin between the two interventions. The GRADE quality of evidence for the 7 relevant outcomes ranged from moderate to very low. 

### 4.2. Comparison with Previous Studies

Similar to the findings of the review by Goldenberg et al. [20] we found moderate evidence for a beneficial effect of a LC diet on weight and BMI at short to intermediate term (3–6 months). However, according to SACN [18], this beneficial effect only occurred at 3 months. Although the quality of evidence for HbA1c in our study was ‘very low’ compared to ‘adequate’ in the SACN review [18] and ‘high’ as reported by Goldenberg et al. [20] all three reviews showed a beneficial effect of a LC diet over the control diet in the short and intermediate term for this glycaemic marker. The short to intermediate term reduction in fasting insulin observed from a LC diet is consistent with the findings of Goldenberg et al. [20] and SACN [18]. In contrast to these two reviews, we observed beneficial effects of a LC diet over LF diet on triglyceride and HDL-C levels, and total cholesterol/HDL-C ratio. The increase in levels of total cholesterol when a LC diet was compared with a LF diet was inconsistent with the findings of Goldenberg et al. [20] who found no effect. Similar to the findings of SACN [18] and Goldenberg et al. [20] we found no significant difference in adverse events between the two diets, but a LC diet resulted in greater reduction in MES compared to a LF diet in our review, whereas our review specifically compared a LC diet with a low LF diet (>26% of total energy or >130 g of carbohydrates a day), the review by Goldenberg et al. [20] compared low and very low carbohydrate diets with a wide range of diets including dietary programs higher in carbohydrates (≥26%), palaeolithic diet as well as no treatment; however, most of the trials included in their review used low fat diets as their control parameters. Furthermore, our review considered only patients with T2D and was based on RCTs, which are the gold standard for evaluating the effectiveness of interventions; whereas, other reviews have included observational studies, people with type 1 diabetes, and a variety of comparator diets [55,56,57,58]. In addition, we evaluated a comprehensive list of cardiometabolic outcomes including blood pressure and markers of renal and liver function which were not evaluated by previous reviews. We also showed that a LC diet was associated with significant reduction in waist circumference at 12 months; an outcome not considered by the reviews of SACN [18] and Goldenberg et al. [20]. 

### 4.3. Explanations for Findings

Dietary protein is associated with greater satiety and therefore reduction in calorie intake [59]. The satiety effect would explain the significantly higher reduction in weight and BMI from the LC compared to LF diet in the short-to-intermediate term. In addition, our findings suggest that weight loss irrespective of carbohydrate and fat restriction resulted in reduction in BMI, serum lipids, and measures of inflammation and glycaemia. Therefore, it was not possible to distinguish between the impact of carbohydrate compared to fat restriction on these outcomes. The improvement in the clinical outcomes may be attributed to calorie-restriction and associated weight loss rather and macronutrient restriction. Thus, a calorie-restricted balanced diet could produce similar favourable clinical outcomes. This is supported by the results from the ongoing DiRECT study (Diabetes Remission Clinical Trial), where a daily calorie intake of 825–853 kcal/day resulted in remission to a non-diabetic state and off antidiabetic drugs for over half the study participants [60]. The short-to-intermediate term beneficial effect of the LC compared to the LF could be due to the difficulty with adherence to the LC, and therefore unsustainable weight loss in the long term. This is also supported by evidence from the DiRECT programme, where sustained remissions at 24 months, was associated with sustained weight loss [7,61]. The DiRECT study involves total meal replacement, stepped food introduction and structured support for long term weight maintenance. Thus, a calorie-restricted balanced diet accompanied by regular behavioural support could be superior to a LC at improving clinical outcomes including remission for people living with T2D.

### 4.4. Implications of Findings

Based on our analysis, a short-to-intermediate term LC diet rather than a LF diet could be recommended for overweight and obese adults with uncontrolled T2M to achieve glycaemic control and weight loss. The LC diet also decreased the requirement for antiglycaemic medications at intermediate-to long-term. Though no adverse findings were observed at long-term, the LC diet may not be beneficial in the long term given no significant evidence of differences between the two diets at 24 months with respect to all outcomes. These findings are in contrast to previous studies, which have reported that LC diets have adverse effects on lipids, blood pressure and renal function [33,34,36,38,45,46,62]. LC diets which are also higher in dietary protein loads cause accumulation of ketones, resulting in abnormal metabolic functioning. However, it should be acknowledged that the literature on the metabolic effects of LC diet comprises heterogenous studies with small sample sizes. By pooling relevant literature on the topic, our findings suggest that LC diets may only be suitable for short term control of glycaemia and weight loss. Given that a LC diet is characterised by the consumption of large amounts of saturated fat and small amounts of fruits, vegetables and fiber, there is a potential for LC diets to adversely impact on lipid profiles, which are major risk factors for coronary heart disease [63]. Indeed, our results showed that LC significantly increased levels of total cholesterol at short-to-intermediate term. Given that patient-centred care is a key aspect of T2D management, [64] patients who choose a LC diet could be supported to manage their diabetes effectively. Patients on this diet should be advised to base their carbohydrates on foods rich in fibre, variety of fruits and vegetables, given their beneficial effect on glycaemic control and cardiometabolic risk factors [65]. In line with general healthy eating advice, limited intake of salt, trans and saturated fats and regular hydration should also be recommended [66].

### 4.5. Strengths and Limitations

Based on evidence from 20 unique RCTs, our review represents an up-to-date comprehensive systematic review and meta-analysis evaluating the efficacy and safety of LC compared with LF diets. Other strengths of the current review included (i) the evaluation of a comprehensive panel of outcomes, which were reported according to time points; (ii) the utilisation of several meta-analytic approaches including ensuring consistency to enhance pooling of most of the data; and (iii) detailed assessment of the risk of bias of included trials and quality of the evidence using the Cochrane risk of bias and GRADE tools, respectively (iv) the evaluation of the effects on blood pressure, renal and liver function, and other markers which were not included in the most recent comprehensive reviews. The limitations were mostly inherent to the studies and included: (i) the inconsistencies in outcome definitions, time points and assessments; (ii) the results of some outcomes were based on single reports; (iii) all trials had a high risk of bias in the domains of blinding of participants and personnel (iv) limited information provided on types of carbohydrates consumed (v) inability to generalise our findings to other populations such as black ethnicities, and (vi) inability to conduct subgroup analysis by relevant characteristics such as age, sex, geographical location, ethnicity, and BMI as prespecified in the protocol, due to the limited studies available for pooling for each outcome and lack of specific data such as ethnic-specific data analyses.

## 5. Conclusions

Except for reducing HbA1c levels and body composition measures at short-to-intermediate term and decreasing the requirement for antiglycaemic medications at intermediate-to-long term, a LC diet appears to be equally effective as a LF diet in terms of control of cardiometabolic markers and the risk of adverse events in obese patients with T2D. The current evidence suggest that LC diets may not be beneficial over the long-term.

## 6. Key Points

Question: Is a low carbohydrate diet more effective for control of cardiometabolic markers and the risk of adverse events in obese patients with T2D compared to a low fat diet?

Findings: Except for reducing HbA1c levels and adiposity parameters at short to intermediate terms, a LC diet appears to be equally effective as a LF diet in terms of control of cardiometabolic markers and the risk of adverse events in obese patients with T2D.

Meaning: A short to intermediate term LC diet could be recommended for overweight and obese adults with uncontrolled T2M to achieve glycaemic control and weight loss.

## Figures and Tables

**Table 1:** Characteristics of included trials.

Author, Year of Publication	Country	Population	Baseline Year	Mean Age, years	Male %	Mean BMI, kg/m^2^	Mean HbA1c, %	Diabetes Duration, years	Trial Duration, years	No. Randomized	No. In Intervention	No. In Comparator	Overall Risk of Bias *
Samaha, 2003 [42]	USA	Obese with diabetes	2001	NR	NR	NR	NR	NR	6.0	79	41	38	High
Daly, 2006 [32]	UK	Obese with poorly controlled T2DM	NR	58.7	48.0	36.1	9.1	NR	3.0	102	51	51	High
Westman, 2008 [50]	USA	Obesity and T2DM	NR	51.8	21.4	38.1	NR	NR	6.0	84	38	46	High
Shai, 2008 [47]	Israel	Obese with T2DM	2005–2007	NR	NR	NR	NR	NR	24.0	31	19	12	High
Davis, 2009 [33]	USA	T2DM with BMI ≥ 25 kg/m^2^, and A1C between 6 and 11%	2004–2006	53.5	21.9	36.0	7.5	NR	12.0	105	55	50	High
Iqbal, 2010 [36]	USA	Obese with T2DM	2004–2008	60.0	89.6	37.5	NR	NR	24.0	144	70	74	High
Goldstein, 2011 [34]	Israel	Obese T2DM	2001–2004	56.0	48.1	33.2	8.9	8.0	12.0	52	26	26	High
Khoo, 2011 [38]	Australia	Obese with T2DM	2007–2008	59.7		35.3		4.5	2.0	31	12	19	High
Guldbrand, 2012 [35]; Jonasson, 2014 [37]	Sweden	Diagnosis of T2DM treated with diet with or without additional oral glucose-lowering medication, incretin-based therapy or insulin	2008–2009	62.0	44.3	32.7	7.3	9.3	24.0	61	30	31	High
Tay, 2014 [48]; Tay 2015 [54]	Australia	Obese adults with T2DM; taking antiglycaemic medication	2012–2013	58.0	57.4	34.6	7.3	8.0	12.0	115	58	57	High
Yamada, 2014 [51]	Japan	Poorly controlled T2DM	2011–2012	63.3	50.0	25.8	7.7	9.2	6.0	24	12	12	High
Saslow, 2014 [43]	USA	Overweight or obese adults with T2DM or prediabetes	2012	59.7	26.5	36.8	6.8	7.1	3.0	34	16	18	High
Sato, 2017 [45]; Sato 2017a [46]	Japan	T2DM with poor glycaemic control	2013–2014	59.4	75.8	26.6	8.2	13.5	18.0	66	33	33	High
Saslow, 2017 [44]	USA	Overweight with T2DM	2013	55.7	40.0		7.2	5.5	7.4	25	12	13	High
Nishimori, 2018 [40]	Japan	T2DM and NFLD	NR	49.5	64.0	NR	NR		3.0	28	14	14	High
Zadeh, 2018 [30]	Iran	Obese and T2DM	NR	48.2		34.1	7.0	6.5	6.0	22	11	11	High
Tay, 2018 [49]	Australia	T2DM under the care of a GP/endocrinologist	2012–2014	58.0	57.4	34.5	7.3	7.0	24.0	115	58	57	High
Perna, 2019 [41]	Italy	Obese and overweight with T2DM; only treated with metformin	NR	67.0	35.3	31.4	6.0	NR	3.0	17	8	9	High
Chen, 2020 [31]	Taiwan	Poorly controlled T2DM	2016	63.6	38.8	NR	NR	9.9	18.0	92	47	45	High
Morris, 2020 [39]	UK	T2D and BMI of at least 30 kg/m^2^	2018	67.0	45.0	35.4	NR	9.2	3.0	33	21	12	High
Gram-Kampmann, 2022 [53]	Denmark	T2DM with HbAIc > 48 mmol/mol	2016–2018	56.6	43.7	NR	NR	5.1	0.5	71	49	22	High
Li, 2022 [52]	China	Overweight or obese with T2DM	2018–2020	36.8	NR	29.4	8.7	0.3	0.25	60	30	30	High

NR—Not Report; T2DM—Type 2 Diabetes Mellitus; NFLD- Non-alcoholic Fatty Liver Disease; GP—General Practitioner; BMI- Body Mass Index; HbA1c—Glycated Haemoglobin; * demonstrated a high risk of bias in one or more domains of the Cochrane risk of bias tool.

**Table 2:** Characteristics of interventions and comparators in included trials.

Author, Year of Publication	Intervention	Description of Intervention	Comparator	Description of Comparator	Exercise Recommendations
Samaha, 2003 [42]	Low carb diet	30 g/day or less	Low fat diet	Caloric restriction sufficient to create a deficit of 500 calories per day, with 30 percent or less of total calories derived from fat.	None recommended
Daly, 2006 [32]	Low carb diet	70 g/day	Low fat diet		Both groups
Westman, 2008 [50]	Low carb ketogenic diet	<20 g of carbohydrate daily	Low-glycemic, reduced-calorie diet	500 kcal/day deficit from weight maintenance diet	Both groups
Shai, 2008 [47]	Low-carb, non–restricted-calorie diet	20 g/day for the 2 month induction phase	Low-fat, restricted-calorie diet	Energy intake of 1500 kcal per day for women and 1800 kcal per day for men, with 30% of calories from fat, 10% of calories from saturated fat, and an intake of 300 mg of cholesterol per day	Not specifically recommended
Davis, 2009 [33]	Low carb diet	Initial 2-week phase of carbohydrate restriction of 20–25 g daily depending on baseline weight; increased intake at 5-g increments each week as participants lost weight	Low fat diet	Fat gram goal was 25% of energy needs, based on baseline weight; 53 energy percent	General recommendations made
Iqbal, 2010 [36]	Low carb diet	<30 g/day	Low fat diet	≤30% of calories from fat with a deficit of 500 kcal/day	Not specifically recommended
Goldstein, 2011 [34]	Modified Atkins diet (very low carb diet)	Containing up to 25 g of carbs daily for the first 6 weeks after randomization, thereafter increasing to a ceiling of 40 g daily	Standard recommended ADA calorie-restricted diet	Containing 10−20% of the daily energy intake from protein and the other 80% divided between fats [which provided 18–20% of calories as MUFA, 8–10% as polyunsaturated fatty acids (PUFA) and 9–10% as SFA], carbohydrates and 35 g of fibre	Both groups
Khoo, 2011 [38]	Low-fat, high-protein, reduced-carb diet	Reduction in daily energy intake by ~600 kcal	Low-calorie diet	1000 kcal/day	Not specifically recommended
Guldbrand, 2012 [35]; Jonasson, 2014 [37]	Low carb diet	20 energy percent from carb	Low fat diet	55–60 energy percent	Not specifically recommended
Tay, 2014 [48]; Tay 2015 [54]	Low-carbohydrate, high unsaturated/low saturated fat diet	14% carbohydrate [<50 g/day], 28% protein, and 58% fat [<10% saturated fat] plus structured exercise	High unrefined carbohydrate, low fat diet	53% carbohydrate, 17% protein, and 30% fat [<10% saturated fat] plus structured exercise	Both groups
Yamada, 2014 [51]	Low carb diet	<130 g/day	Calorie restricted diet	Carbohydrates = 50–60%, protein = 1.0–1.2 g/kg (<20%) and fat = <25%	Not reported
Saslow, 2014 [43]	Low carb ketogenic diet	Reduce carbohydrate intake over 7–10 days to between 20–50 g of carbohydrates a day with the goal of achieving nutritional ketosis	Moderate carb, calorie-restricted diet	45% to 50% of calories derived from carbohydrates	Both groups
Sato, 2017 [45]; Sato 2017a [46]	Low carb diet	130 g/day	Calorie restricted diet	The percentage of carbohydrate per total calorie was 50–60%, and that of proteinwas 1.0–1.2 g/kg	
Saslow, 2017 [44]	Very low-carb ketogenic diet with lifestyle factors; “intervention”	Reduce carb intake to between 20–50 g of nonfiber carbohydrates a day	Online diet program based around a plate method diet	A low-fat diet that emphasizes green vegetables, lean protein sources, and somewhat limited starchy and sweet foods	Intervention group received exercise recommendations
Nishimori, 2018 [40]	Low carb diet	70–130 g/day	Calorie restricted diet	25 kcal/kg of ideal body weight per day	Not reported
Zadeh, 2018 [30]	Low carb diet plus high intensity interval training	45% energy (E %) from fat, 20 E% from carbohydrate and 35 E% from protein	Low fat diet plus high intensity interval training	30 E% from fat (less than 10 E% from saturated fat), 50 E% from carbohydrate and 20 E% from protein	Both groups
Tay, 2018 [49]	Low-carb, high-unsaturated/low-saturated fat diet	14% energy as carb, 28% as protein, 58% as fat (<10% saturated fat)	High-carbohydrate, low-fat diet	53% as CHO, 17% as protein, 30% as fat (<10% saturated fat)	Both groups
Perna, 2019 [41]	Low carb diet and metformin	CHO <125 g/ day, 1600 kcal/day for females and 1800 kcal/day for males	Balanced standard diet	Carbohydrates 55–60%, lipids 25–30%, proteins 15–20%	Not reported
Chen, 2020 [31]	Low carb diet	90 g/day	Traditional diabetic diet	Macronutrient percentage was 50–60% for CHO, 1.0–1.2 g/kg for protein and ≤30% for fat	Both groups
Morris, 2020 [39]	Low-energy, low-carb diet	800–1000 kcal/day, with <26% of daily energy intake from carb and a minimum of 60 g protein/day	Usual care dietary advice	Healthy balanced eating	Not reported
Gram-Kampmann, 2022 [53]	Low carb diet	Maximum of 20 E% of carbohydrates (mainly complex and water-soluble), 50–60 E% fat, and 25–30 E% protein.	Control diet	50–60 E% carbohydrates mainly from fruit, vegetables, and whole-grain sources, 20–30 E% fat	Both groups
Li, 2022 [52]	Ketogenic diet	carbohydrate 30–50 g, protein 60 g, fat 130 g, and total calories (1500 ± 50) Kcal	Routine diet for diabetes	Carbohydrate 250–280 g, protein 60 g, fat 20 g, total calories (1500 ± 50) Kcal	Not reported

## Data Availability

Not applicable.

## Supplementary Materials

The following supporting information can be downloaded at: https://www.mdpi.com/article/10.3390/nu14204391/s1, Table S1. PRISMA checklist. Table S2. MOOSE checklist. Table S3. Literature search strategy. Table S4. GRADE summary of findings. Figure S1. Risk of bias assessment. Figure S2. Low carbohydrate versus low fat diet and fat free mass. Figure S3. Low carbohydrate versus low fat diet and diastolic blood pressure. Figure S4. Low carbohydrate versus low fat diet and total cholesterol. Figure S5. Low carbohydrate versus low fat diet and LDL-cholesterol. Figure S6. Low carbohydrate versus low fat diet and HDL-cholesterol. Figure S7. Low carbohydrate versus low fat diet and triglycerides. Figure S8. Low carbohydrate versus low fat diet and total cholesterol/HDL-C ratio. Figure S9. Low carbohydrate versus low fat diet and C-reactive protein. Figure S10. Low carbohydrate versus low fat diet and IL-6. Figure S11. Low carbohydrate versus low fat diet and fasting insulin. Figure S12. Low carbohydrate versus low fat diet and HOMA2-IR. Figure S13. Low carbohydrate versus low fat diet and HOMA-IR. Figure S14. Low carbohydrate versus low fat diet and HOMA2-%B. Figure S15. Low carbohydrate versus low fat diet and ALT. Figure S16. Low carbohydrate versus low fat diet and AST. Figure S17. Low carbohydrate versus low fat diet and GGT. Figure S18. Low carbohydrate versus low fat diet and creatinine. Figure S19. Low carbohydrate versus low fat diet and estimated GFR. Figure S20. Low carbohydrate versus low fat diet and microalbumin. Figure S21. Low carbohydrate versus low fat diet and urea. Figure S22. Low carbohydrate versus low fat diet and urinary albumin. Figure S23. Low carbohydrate versus low fat diet and uric acid. Figure S24. Low carbohydrate versus low fat diet and medication effect score. Figure S25. Low carbohydrate versus low fat diet and risk of adverse events.
