# Supplementary material for: Comparison of the Effectiveness of Low Carbohydrate Versus Low Fat Diets, in Type 2 Diabetes: Systematic Review and Meta-Analysis of Randomized Controlled Trials
Source: Nutrients. 2022 Oct 19;14(20):4391. doi: 10.3390/nu14204391 (PMC9609579; doi:10.3390/nu14204391)
Supplement: Supplementary file 1 [file nutrients-14-04391-s001.zip › nutrients-1957739-supplementary.pdf]

Table S1. PRISMA checklist

Section/topic	Item No	Checklist item	Reported on page No
**Title**			
Title	1	Identify the report as a systematic review, meta-analysis, or both	1
**Abstract**			
Structured summary	2	Provide a structured summary including, as applicable, background, objectives, data sources, study eligibility criteria, participants, interventions, study appraisal and synthesis methods, results, limitations, conclusions and implications of key findings, systematic review registration number	2
**Introduction**			
Rationale	3	Describe the rationale for the review in the context of what is already known	Introduction
Objectives	4	Provide an explicit statement of questions being addressed with reference to participants, interventions, comparisons, outcomes, and study design (PICOS)	Introduction
**Methods**			
Protocol and registration	5	Indicate if a review protocol exists, if and where it can be accessed (such as web address), and, if available, provide registration information including registration number	Methods
Eligibility criteria	6	Specify study characteristics (such as PICOS, length of follow-up) and report characteristics (such as years considered, language, publication status) used as criteria for eligibility, giving rationale	Methods
Information sources	7	Describe all information sources (such as databases with dates of coverage, contact with study authors to identify additional studies) in the search and date last searched	Methods
Search	8	Present full electronic search strategy for at least one database, including any limits used, such that it could be repeated	Supplementary Material 3
Study selection	9	State the process for selecting studies (that is, screening, eligibility, included in systematic review, and, if applicable, included in the meta-analysis)	Methods
Data collection process	10	Describe method of data extraction from reports (such as piloted forms, independently, in duplicate) and any processes for obtaining and confirming data from investigators	Methods
Data items	11	List and define all variables for which data were sought (such as PICOS, funding sources) and any assumptions and simplifications made	Methods
Risk of bias in individual studies	12	Describe methods used for assessing risk of bias of individual studies (including specification of whether this was done at the study or outcome level), and how this information is to be used in any data synthesis	Methods
Summary measures	13	State the principal summary measures (such as risk ratio, difference in means).	Methods
Synthesis of results	14	Describe the methods of handling data and combining results of studies, if done, including measures of consistency (such as I^2 statistic) for each meta-analysis	Methods
Risk of bias across studies	15	Specify any assessment of risk of bias that may affect the cumulative evidence (such as publication bias, selective reporting within studies)	Methods
Additional analyses	16	Describe methods of additional analyses (such as sensitivity or subgroup analyses, meta-regression), if done, indicating which were pre-specified	Methods
**Results**			
Study selection	17	Give numbers of studies screened, assessed for eligibility, and included in the review, with reasons for exclusions at each stage, ideally with a flow diagram	Results and Figure 1
Study characteristics	18	For each study, present characteristics for which data were extracted (such as study size, PICOS, follow-up period) and provide the citations	Results; Table 1
Risk of bias within studies	19	Present data on risk of bias of each study and, if available, any outcome-level assessment (see item 12).	Results; Table 1; SM 4
Results of individual studies	20	For all outcomes considered (benefits or harms), present for each study (a) simple summary data for each intervention group and (b) effect estimates and confidence intervals, ideally with a forest plot	Results; Figures 2-5; SM 5-29
Synthesis of results	21	Present results of each meta-analysis done, including confidence intervals and measures of consistency	Results; Figures 2-5; SM 5-29
Risk of bias across studies	22	Present results of any assessment of risk of bias across studies (see item 15)	SM 4
Additional analysis	23	Give results of additional analyses, if done (such as sensitivity or subgroup analyses, meta-regression) (see item 16)	NA
**Discussion**			
Summary of evidence	24	Summarise the main findings including the strength of evidence for each main outcome; consider their relevance to key groups (such as health care providers, users, and policy makers)	Discussion
Limitations	25	Discuss limitations at study and outcome level (such as risk of bias), and at review level (such as incomplete retrieval of identified research, reporting bias)	Discussion
Conclusions	26	Provide a general interpretation of the results in the context of other evidence, and implications for future research	Discussion
**Funding**			
Funding	27	Describe sources of funding for the systematic review and other support (such as supply of data) and role of funders for the systematic review	After discussion

Table S2. MOOSE checklist

Comparison of the effectiveness of low carbohydrate versus low fat diets, in type 2 diabetes. Systematic review and meta-analysis of randomised controlled trials

Criteria		Brief description of how the criteria were handled in the review
**Reporting of background**		
√	Problem definition	The net clinical benefit of low carbohydrate diets compared with low fat diets for people with type 2 diabetes (T2D) remains uncertain
√	Hypothesis statement	There are no differences in the efficacy and safety of low carbohydrate compared with low fat diets in people with T2D.
√	Description of study outcomes	Measures of glycaemia; body composition; cardiovascular risk markers; liver function tests; renal function tests; measures of medication changes; and adverse events
√	Type of exposure	Low carbohydrate and low fat diets
√	Type of study designs used	Randomised controlled trials (RCTs)
√	Study population	T2D and prediabetes
**Reporting of search strategy should include**		
√	Qualifications of searchers	Tanefa Apekey, PhD; Setor K. Kunutsor, PhD
√	Search strategy, including time period included in the synthesis and keywords	Time period: from inception to July 2021<br>The detailed search strategy can be found in Supplementary Material 3
√	Databases and registries searched	MEDLINE, Embase, Web of Science, and Cochrane databases
√	Search software used, name and version, including special features	OvidSP was used to search EMBASE and MEDLINE<br>EndNote used to manage references
√	Use of hand searching	We searched bibliographies of retrieved papers
√	List of citations located and those excluded, including justifications	Details of the literature search process are outlined in the flow chart. The citation list for excluded studies are available on request.
√	Method of addressing articles published in languages other than English	Not applicable
√	Method of handling abstracts and unpublished studies	Abstracts with no full text publications were not included.
√	Description of any contact with authors	None
**Reporting of methods should include**		
√	Description of relevance or appropriateness of studies assembled for assessing the hypothesis to be tested	Detailed inclusion and exclusion criteria are described in the Methods section.
√	Rationale for the selection and coding of data	Data extracted from each of the studies were relevant to the population characteristics, study design, exposure, and outcome.
√	Assessment of confounding	Not applicable
√	Assessment of study quality, including blinding of quality assessors; stratification or regression on possible predictors of study results	Risk of bias in RCTs was assessed using the Cochrane Risk of Bias Tool
√	Assessment of heterogeneity	Heterogeneity of the studies was quantified with I^2 statistic that provides the relative amount of variance of the summary effect due to the between-study heterogeneity
√	Description of statistical methods in sufficient detail to be replicated	Description of methods of meta-analyses. We performed random effects meta-analysis with Stata 16.
√	Provision of appropriate tables and graphics	Table 1; Figures 1-5; Supplementary Materials 4-29
**Reporting of results should include**		
√	Graph summarizing individual study estimates and overall estimate	Figures 2-5; Supplementary Materials 5-29
√	Table giving descriptive information for each study included	Table 1
√	Results of sensitivity testing	Not applicable
√	Indication of statistical uncertainty of findings	95% confidence intervals were presented with all summary estimates, I^2 values and results of sensitivity analyses
**Reporting of discussion should include**		
√	Quantitative assessment of bias	Risk of bias assessment discussed. GRADE quality of evidence reported.
√	Justification for exclusion	All studies were excluded based on the pre-defined inclusion criteria in methods section.

√	Assessment of quality of included studies	Brief discussion included in ‘Methods’ section
**Reporting of conclusions should include**		
√	Consideration of alternative explanations for observed results	Discussion
√	Generalization of the conclusions	Discussed in the context of the results.
√	Guidelines for future research	Large-scale definitive trials warranted
√	Disclosure of funding source	In “Acknowledgement” section

Table S3. Literature search strategy

Relevant studies, published from inception to July 2021 (date last searched), were identified through electronic searches using PubMed, MEDLINE, Embase, Web of Science, Clinical Trials.gov, and the Cochrane electronic databases. Electronic searches were supplemented by scanning reference lists of articles identified for all relevant studies (including review articles) and by hand searching of relevant journals.

(i) MEDLINE strategy to identify relevant diet exposures:
"diet, carbohydrate-restricted"[MeSH Terms] OR carbohydrate restricted diet[Text Word]
("diet"[MeSH Terms] OR diet[Text Word]) AND ("carbohydrates"[MeSH Terms] OR carbohydrate[Text Word]) AND restricted[All Fields]
"diet, high-protein"[MeSH Terms] OR high protein diet[Text Word]
"diet, fat-restricted"[MeSH Terms] OR low fat diet[Text Word]
"diet, fat-restricted"[MeSH Terms] OR fat restricted diet[Text Word]
"diet, ketogenic"[MeSH Terms]

(ii) MEDLINE strategy to identify relevant outcomes:
"diabetes mellitus, type 2"[MeSH Terms] OR type 2 diabetes[Text Word]
"diabetes mellitus"[MeSH Terms] OR diabetes mellitus[Text Word]
T2D[All Fields]
"insulin resistance"[MeSH Terms] OR insulin resistant[Text Word]
"metabolic syndrome"[MeSH Terms] OR "insulin resistance"[MeSH Terms] OR Insulin Resistance syndrome[Text Word]
"glycated hemoglobin a"[MeSH Terms] OR HbA1c[Text Word]

(iii) MEDLINE strategy to identify relevant population:
("humans"[MeSH Terms])

Parts i, ii and iii were combined using ‘AND’ to search MEDLINE. Each part was specifically translated for searching alternative databases.

Figure S1. Risk of bias assessment

	Randon sequence generation	Allocation concealment	Blinding of participants & personnel	Blinding of outcome assessments	Incomplete outcome data	Selective reporting	Other bias
Samaha, 2003	+	+	-	-	?	?	+
Daly, 2006	+	+	-	-	-	+	+
Westman, 2008	+	+	-	-	+	+	+
Shai, 2008	+	+	-	-	?	?	+
Davis, 2009	+	+	-	-	+	+	+
Iqbal, 2010	+	-	-	-	+	+	+
Goldstein, 2011	?	-	-	-	+	+	+
Khoo, 2011	?	-	-	-	+	+	+
Guldbrand, 2012; Jonasson, 2014	+	+	-	-	+	+	+
Tay, 2014; Tay 2015	+	+	-	+	+	+	+
Yamada, 2014	+	?	-	-	+	+	+
Saslow, 2014	+	+	-	-	+	+	+
Sato, 2017; Sato 2017a	+	+	-	-	+	+	+
Saslow, 2017	+	+	-	-	?	-	?
Nishimori, 2018	+	+	-	-	+	+	+
Zadeh, 2018	+	+	-	-	+	+	+
Tay, 2018	+	+	-	+	+	+	+
Perna, 2019	?	-	-	-	+	+	+
Chen, 2020	+	?	-	-	+	+	+
Morris, 2020	+	+	-	-	+	+	+
Gram-Kampmann, 2021	+	+	-	-	+	+	?
Li, 2022	?	-	-	-	?	+	?

+	**Low risk of bias**
?	**Unclear risk of bias**
-	**High risk of bias**

Figure S2. Low carbohydrate versus low fat diet and fat free mass

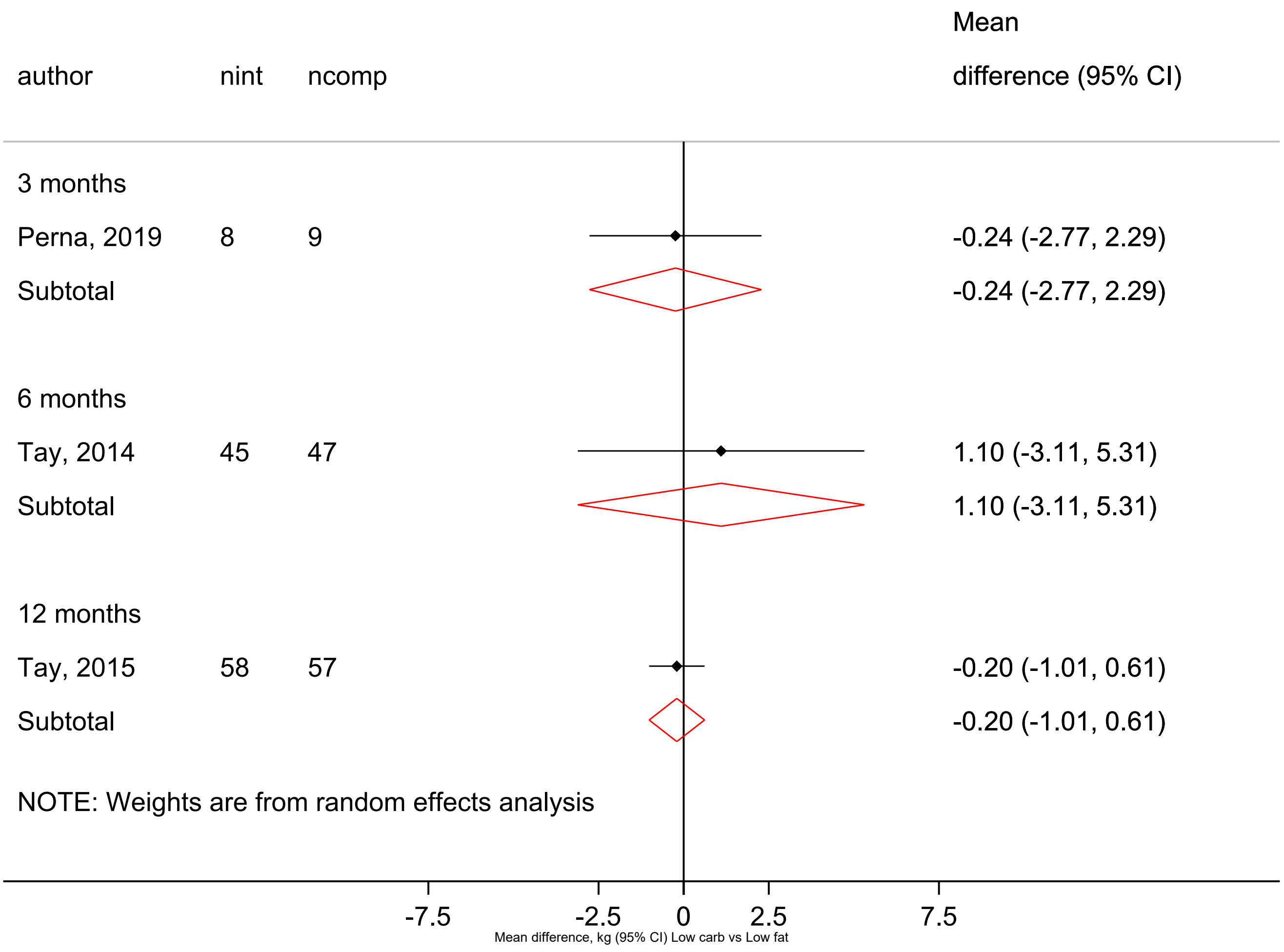

CI, confidence interval (bars)

Figure S3. Low carbohydrate versus low fat diet and diastolic blood pressure

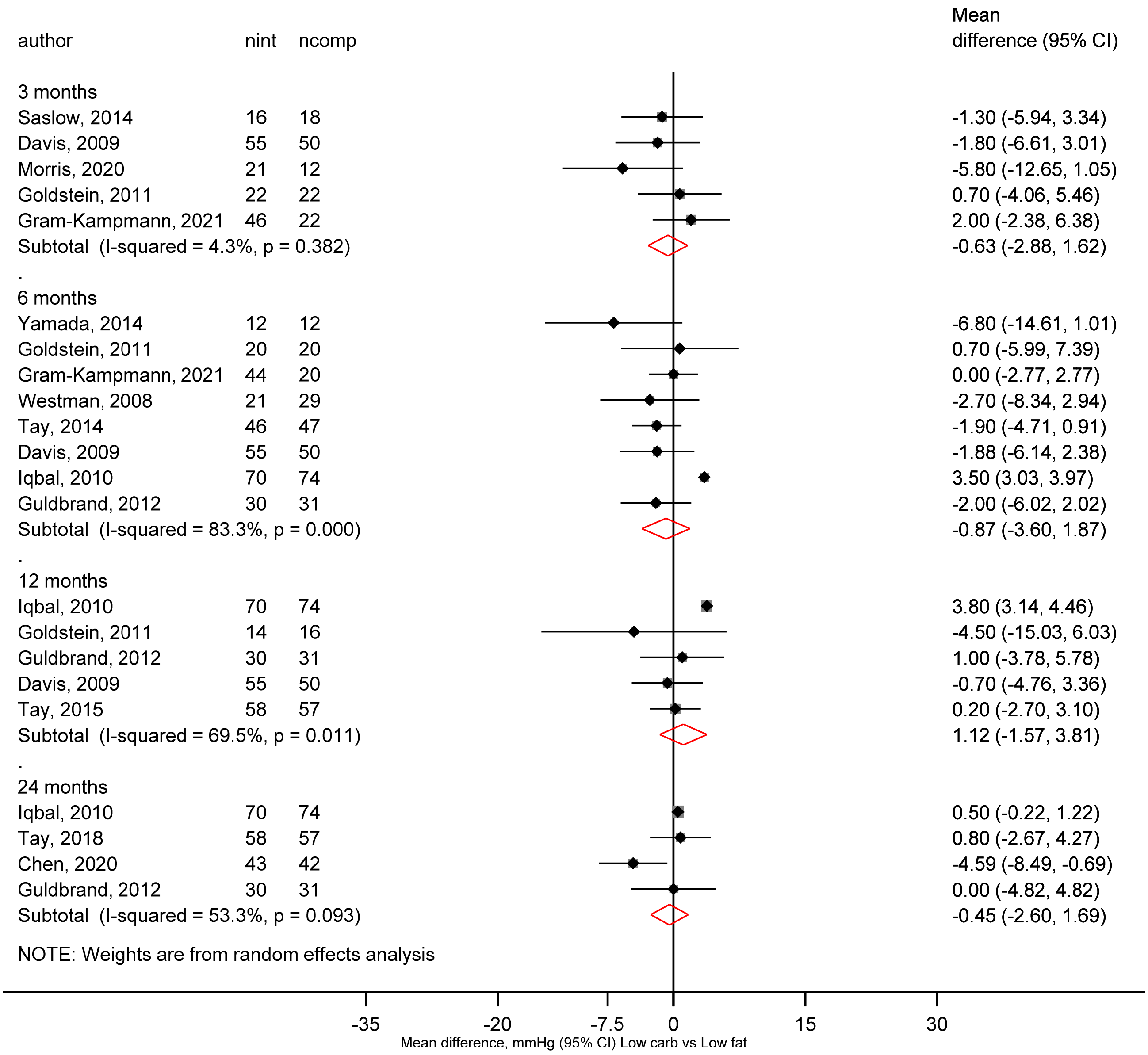

CI, confidence interval (bars)

Figure S4. Low carbohydrate versus low fat diet and total cholesterol

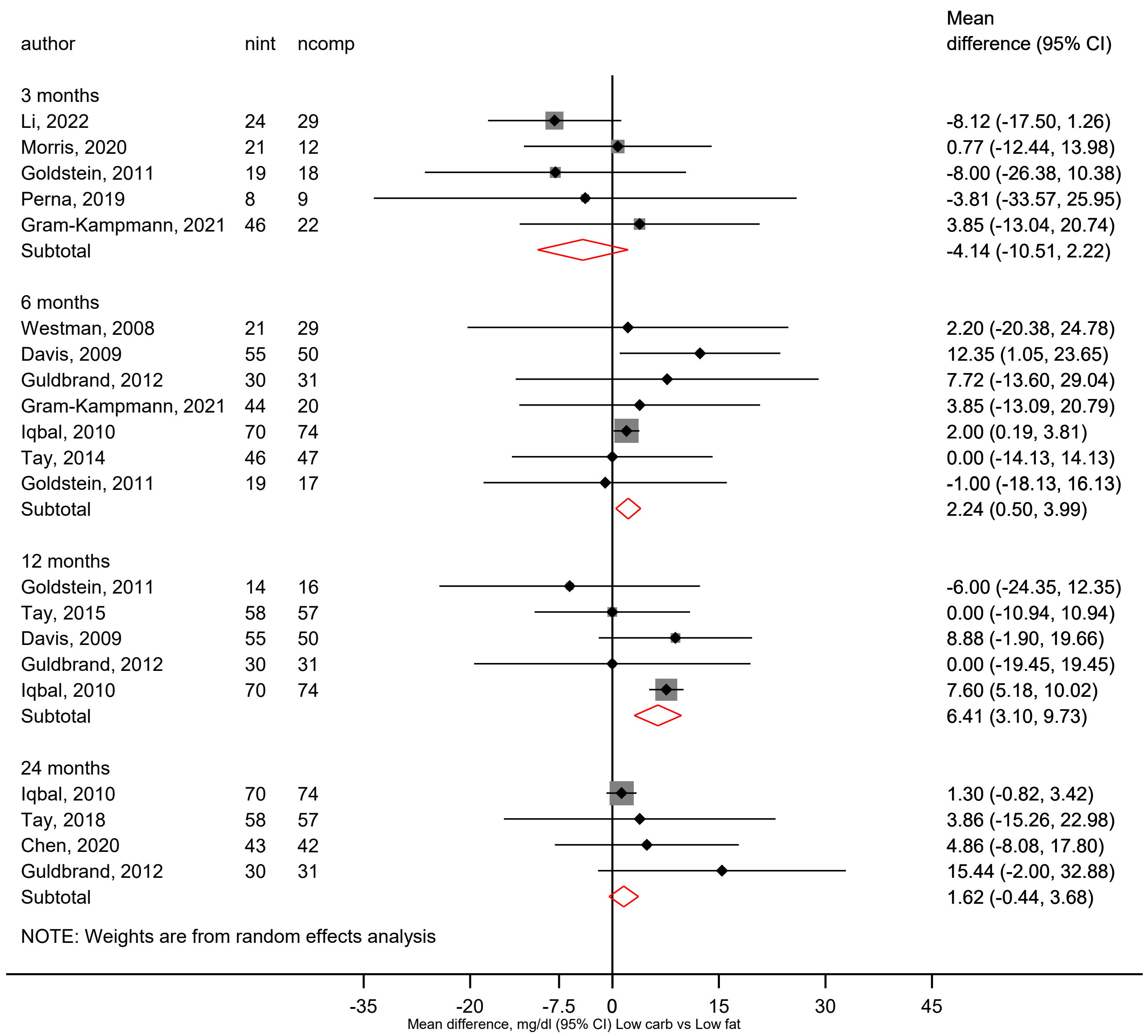

CI, confidence interval (bars)

Figure S5. Low carbohydrate versus low fat diet and LDL-cholesterol

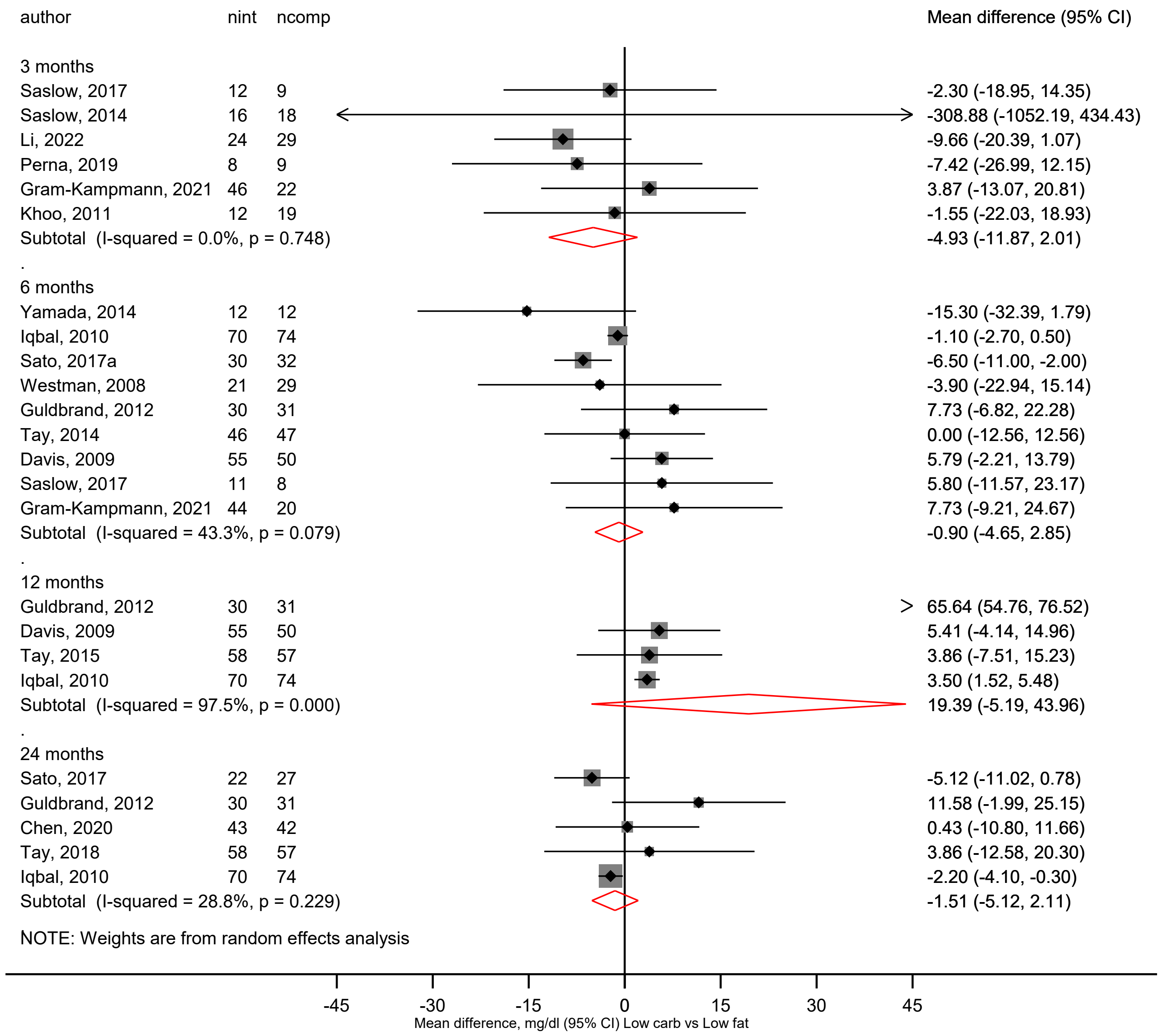

CI, confidence interval (bars); LDL, low-density cholesterol

Figure S6. Low carbohydrate versus low fat diet and HDL-cholesterol

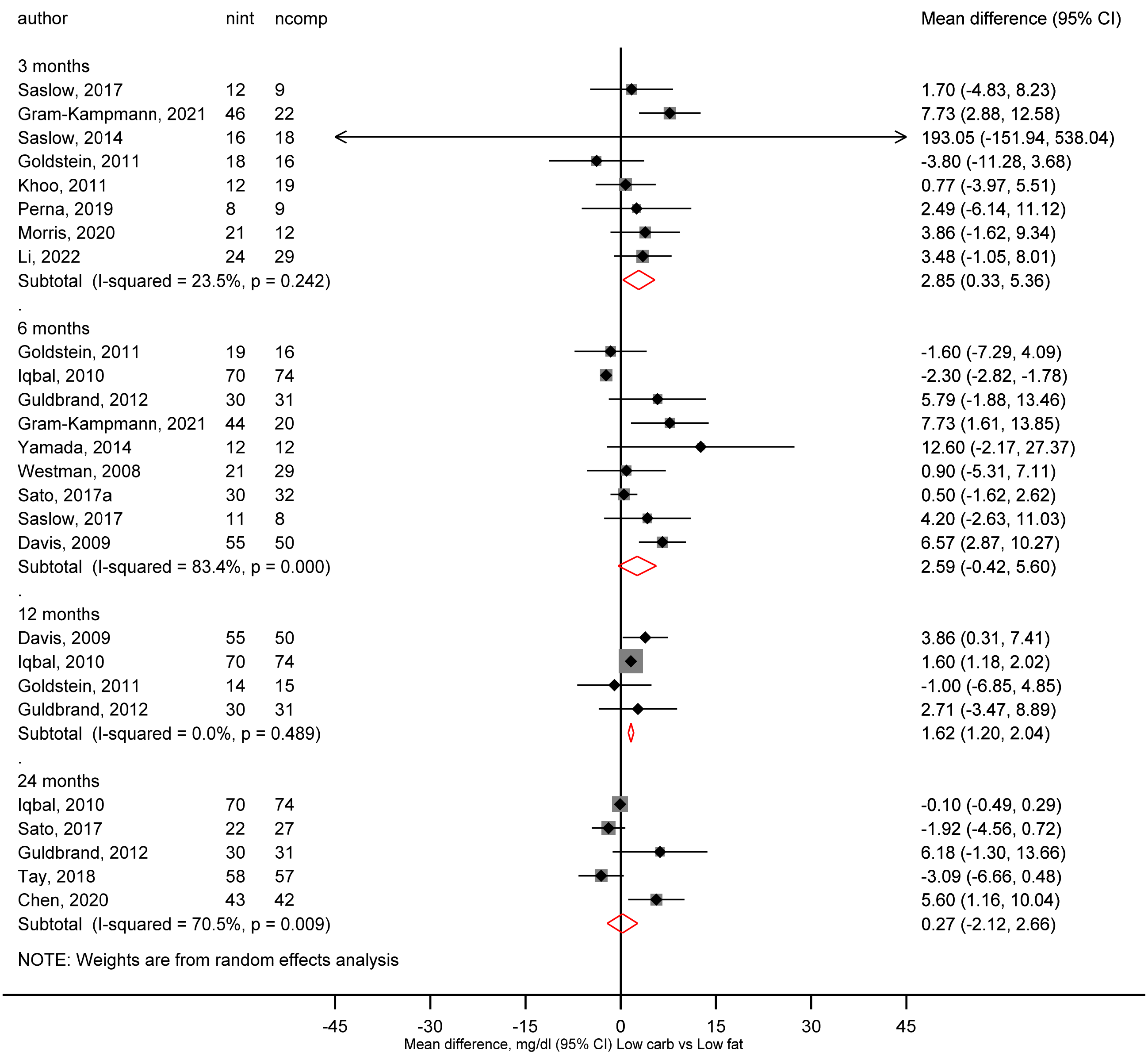

CI, confidence interval (bars); HDL, high-density cholesterol

Figure S7. Low carbohydrate versus low fat diet and triglycerides

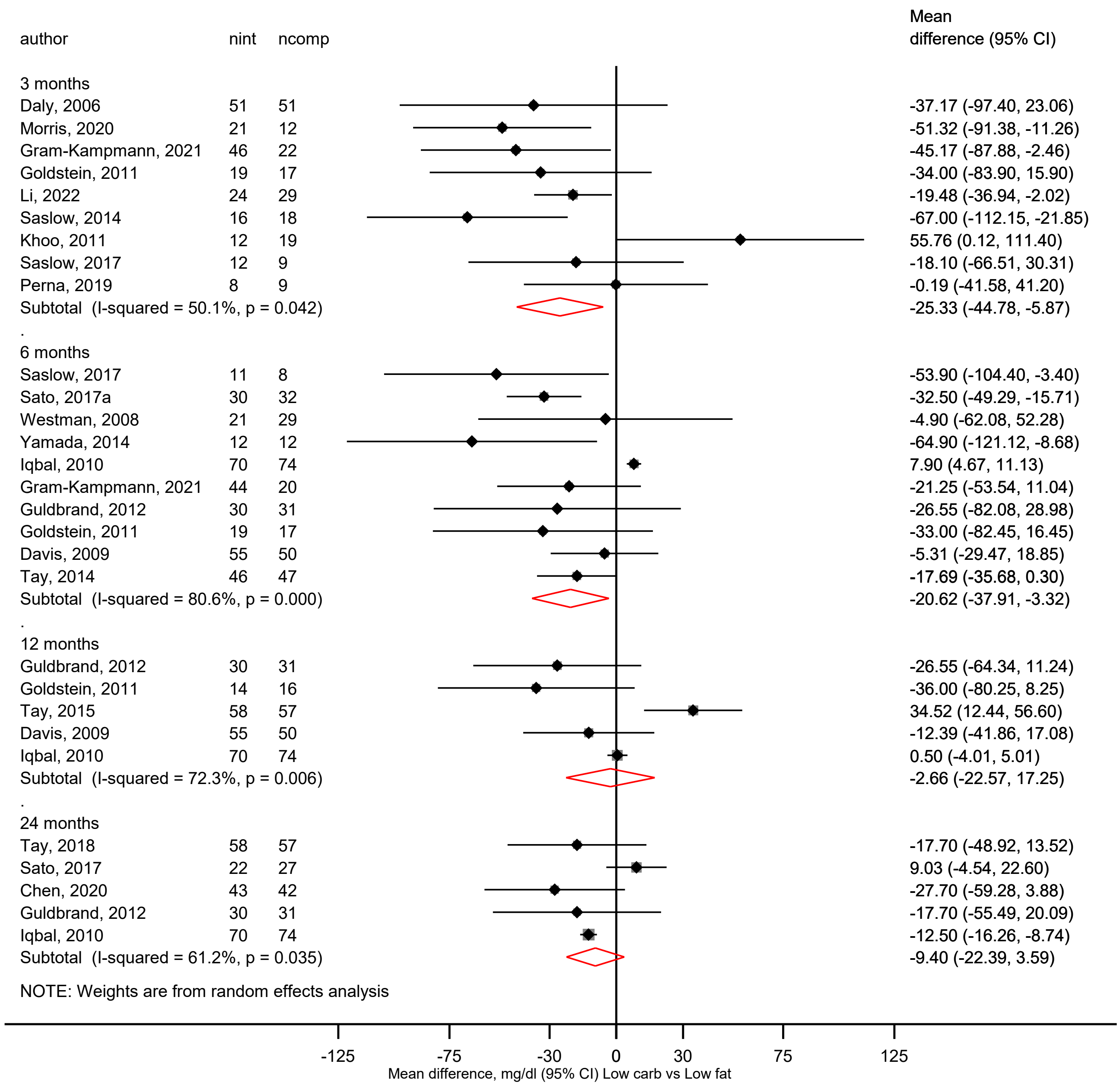

CI, confidence interval (bars)

Figure S8. Low carbohydrate versus low fat diet and total cholesterol/HDL-C ratio

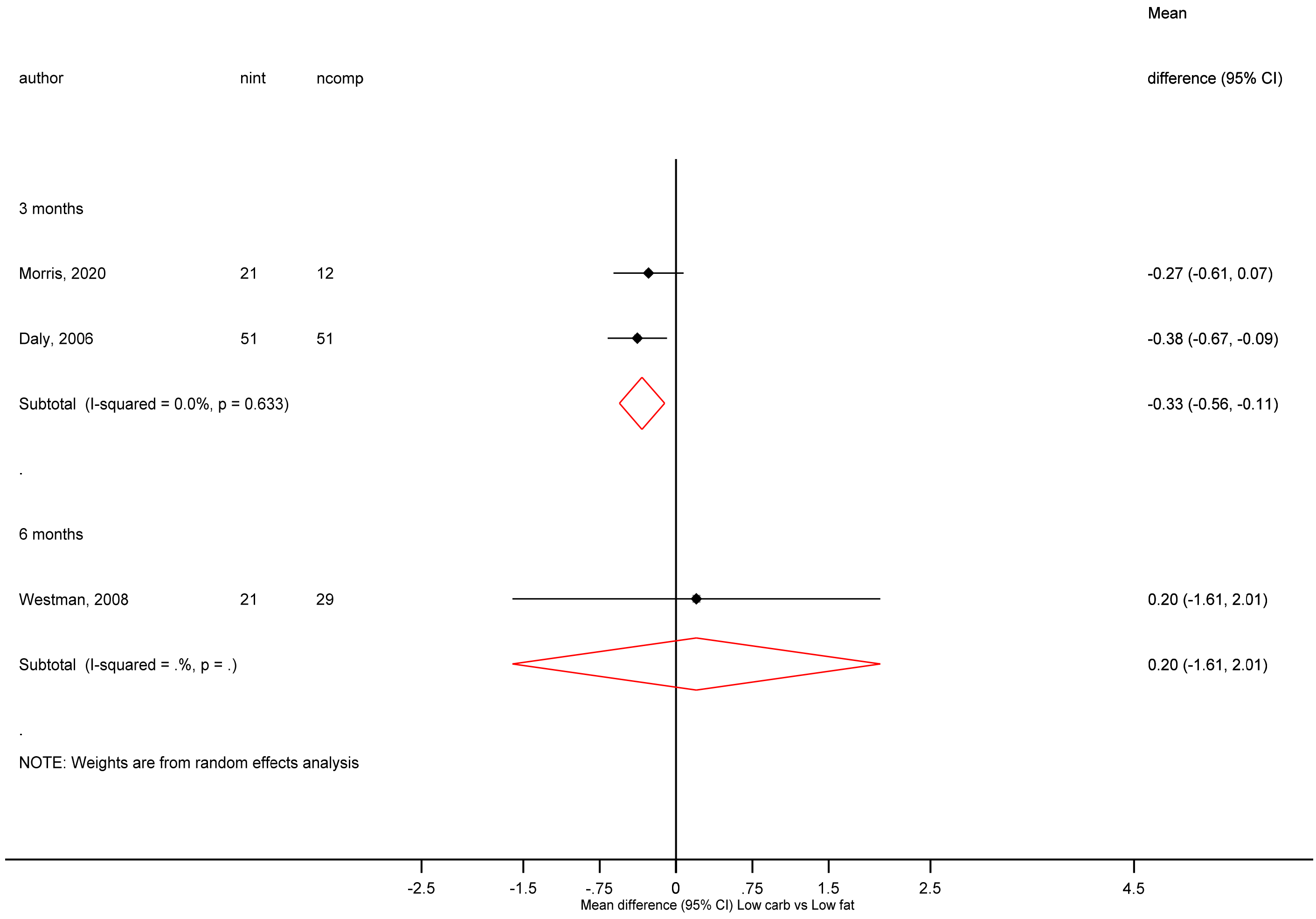

CI, confidence interval (bars); HDL, high-density cholesterol

Figure S9. Low carbohydrate versus low fat diet and C-reactive protein

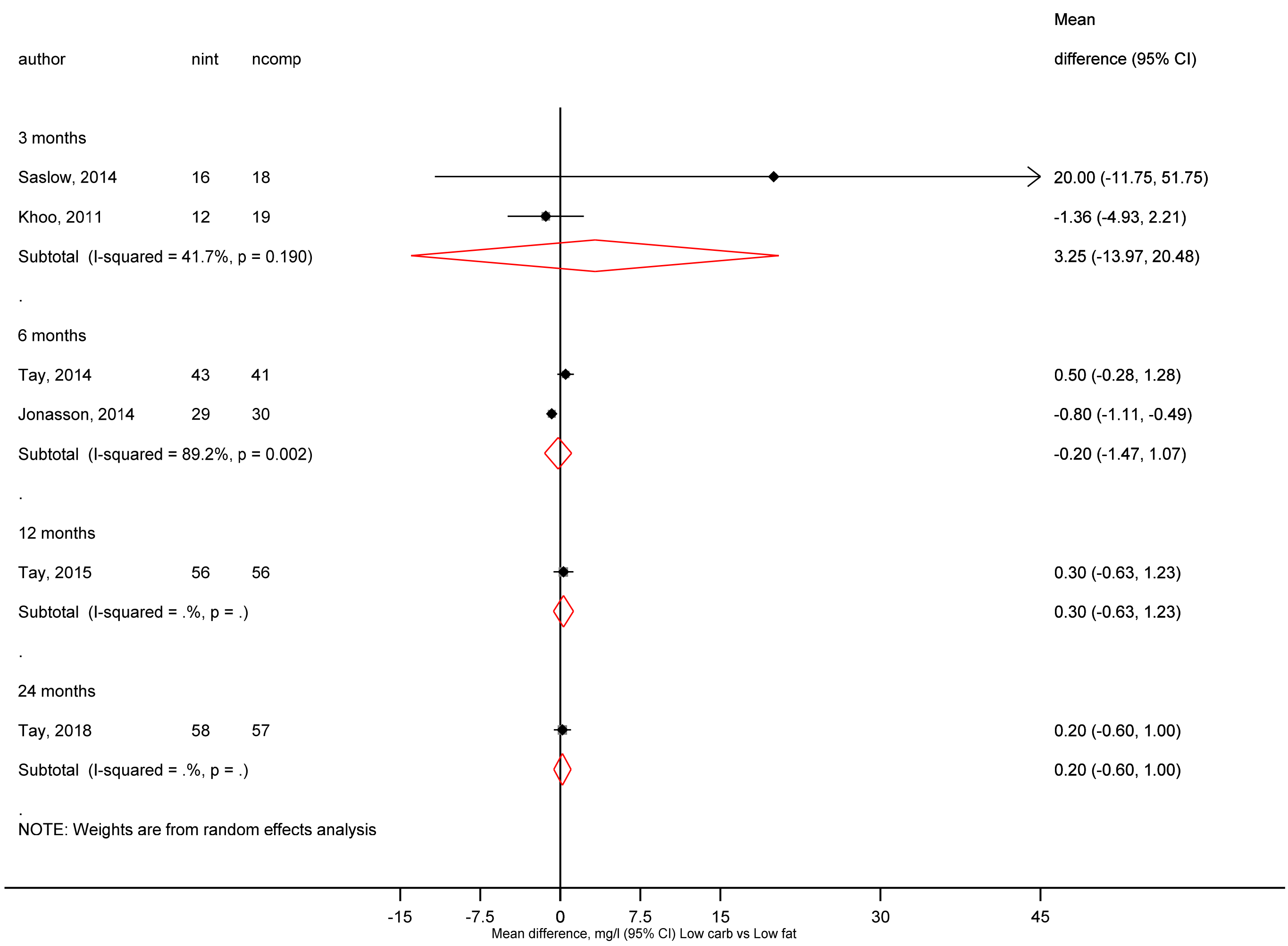

CI, confidence interval (bars)

Figure S10. Low carbohydrate versus low fat diet and IL-6

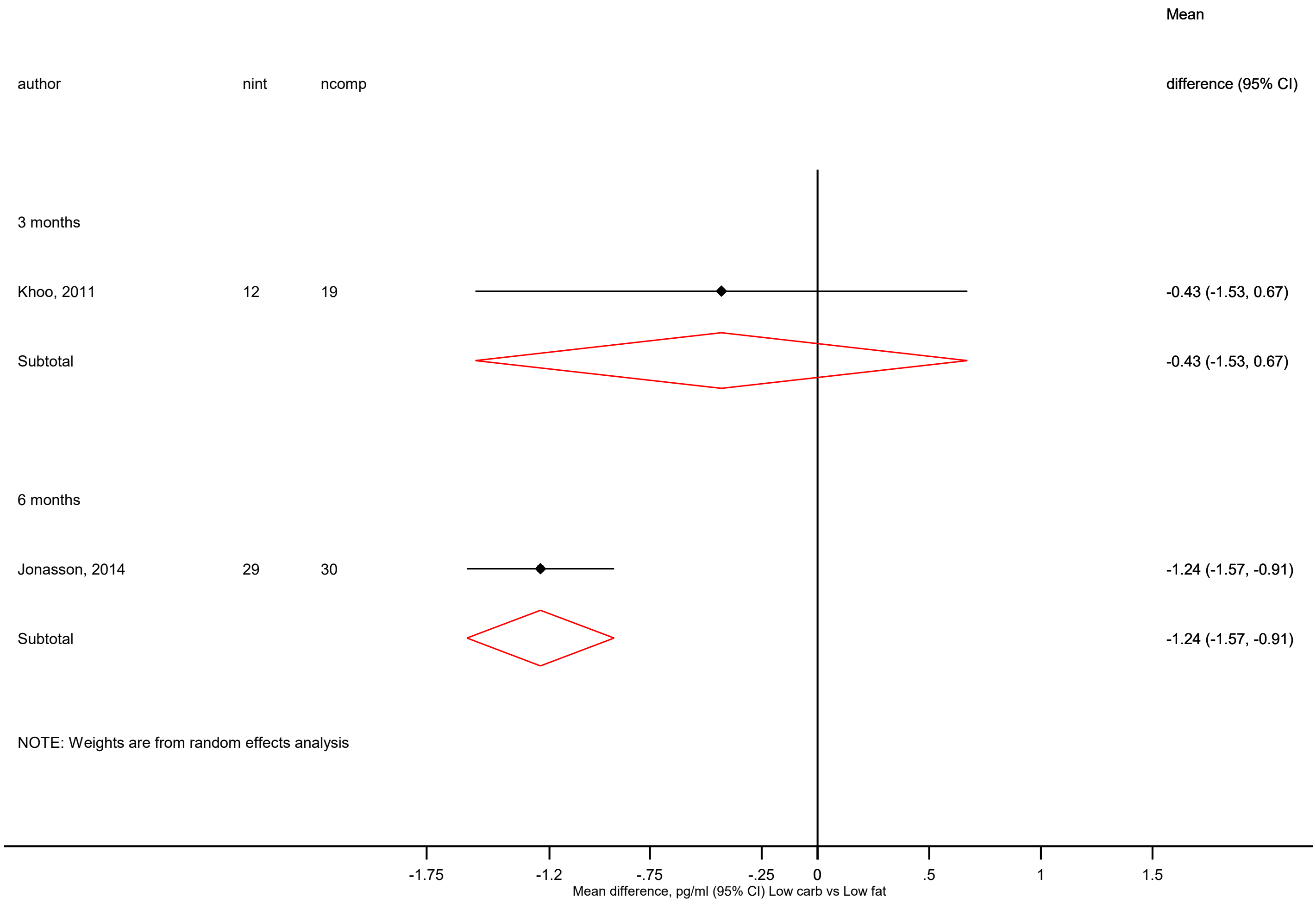

CI, confidence interval (bars); IL-6, interleukin-6

Figure S11. Low carbohydrate versus low fat diet and fasting insulin

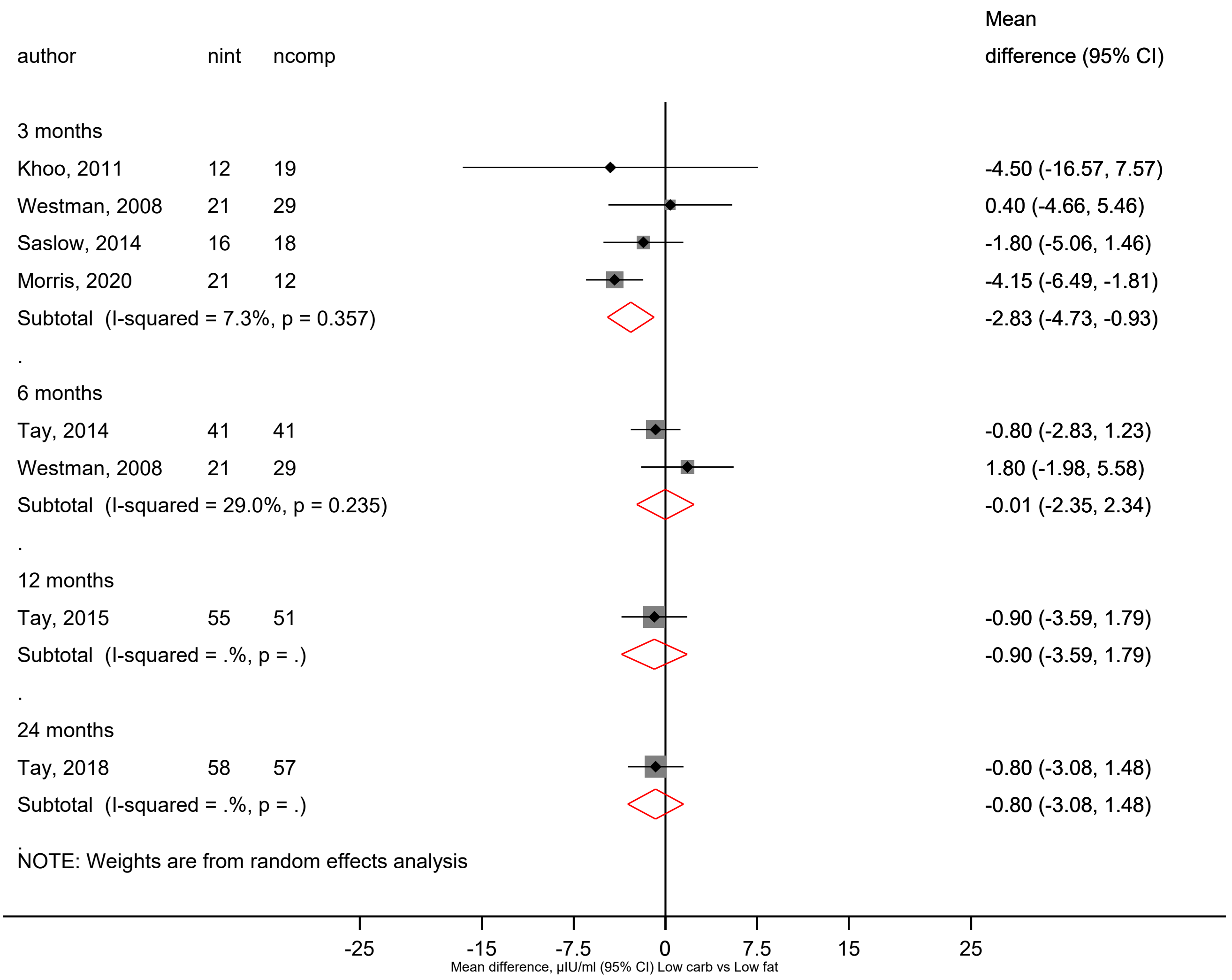

CI, confidence interval (bars)

Figure S12. Low carbohydrate versus low fat diet and HOMA2-IR

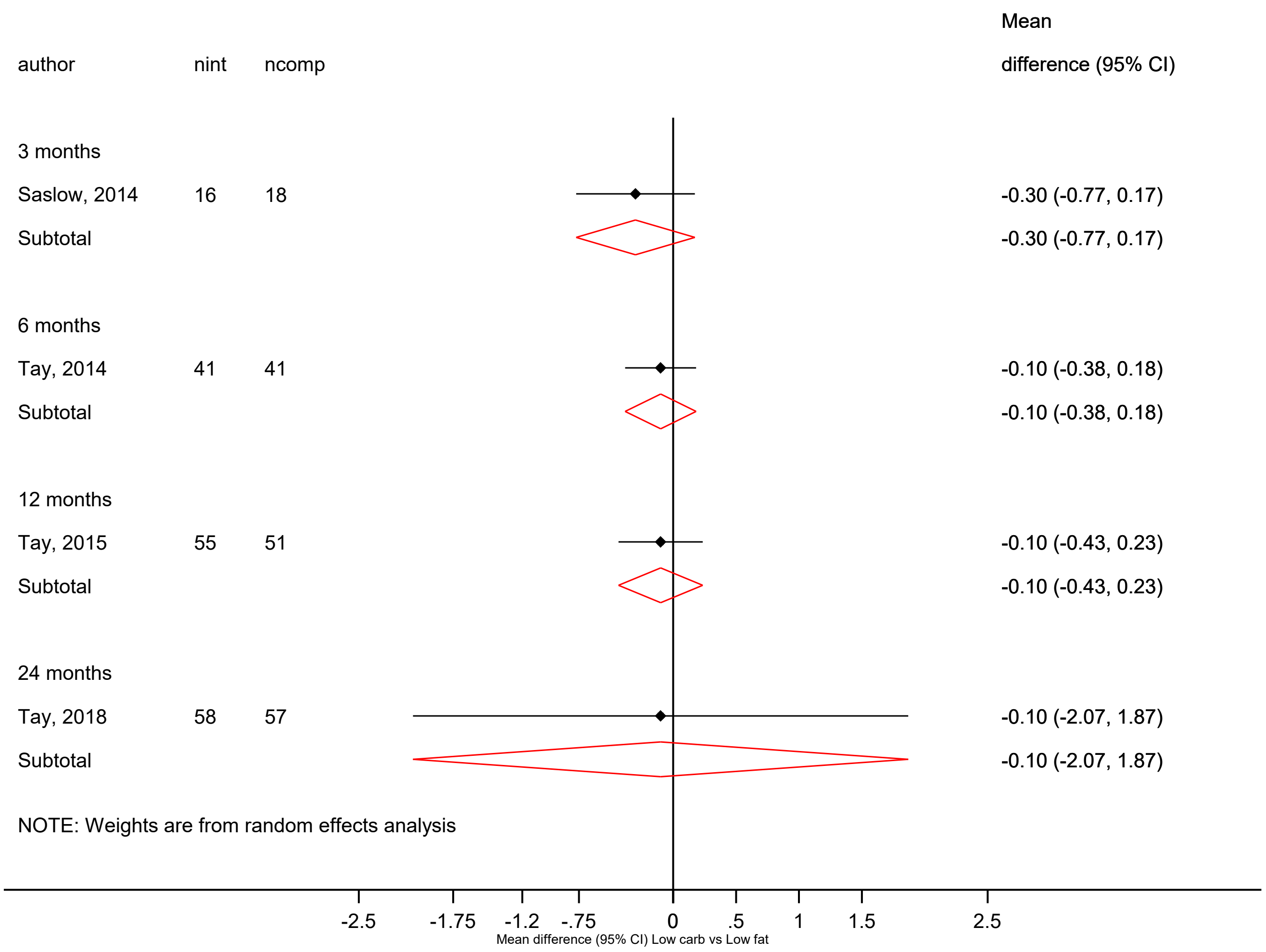

CI, confidence interval (bars); HOMA2-IR, homeostasis model assessment 2-insulin resistance

Figure S13. Low carbohydrate versus low fat diet and HOMA-IR

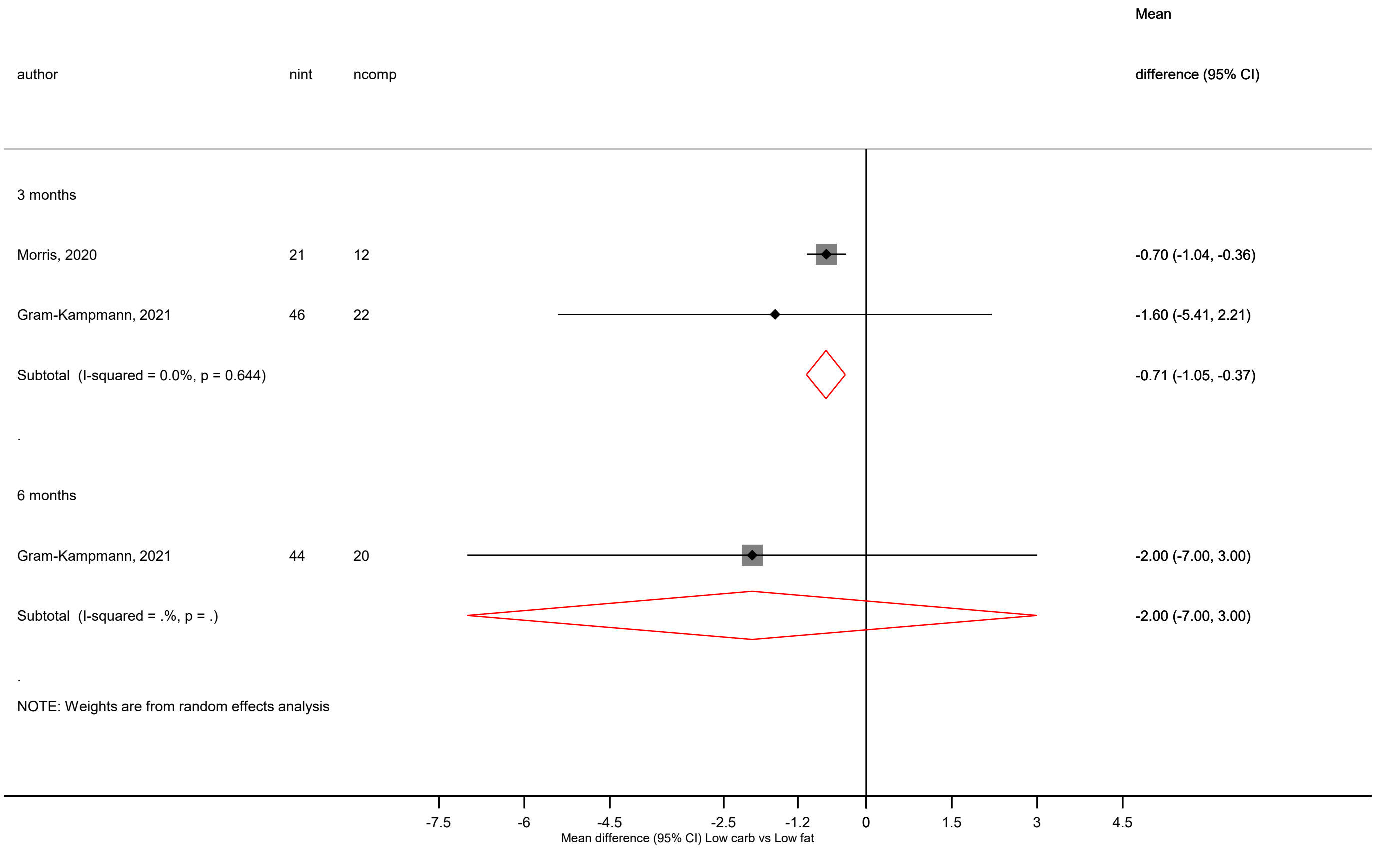

CI, confidence interval (bars); HOMA-IR, homeostasis model assessment-insulin resistance

Figure S14. Low carbohydrate versus low fat diet and HOMA2-%B

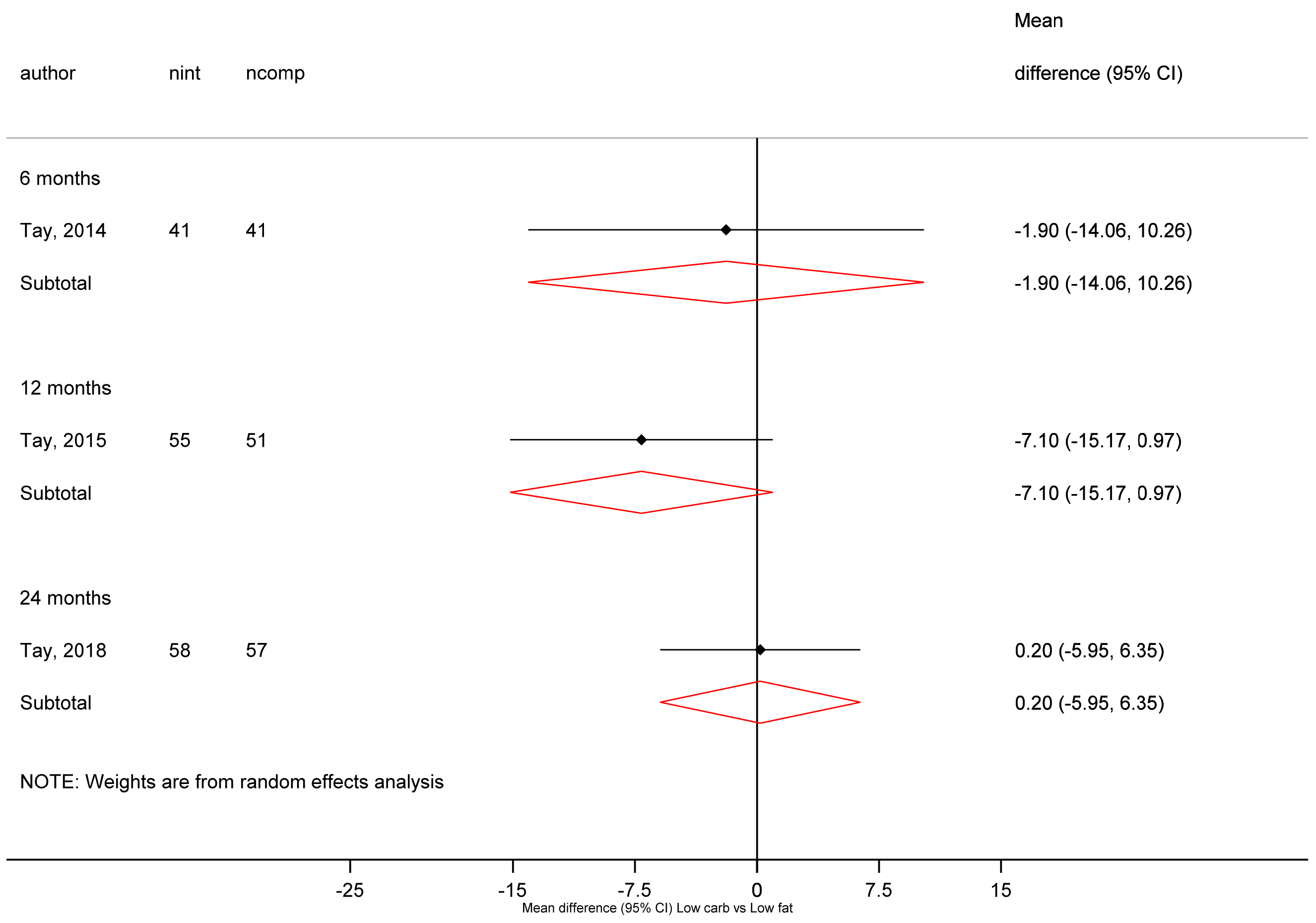

CI, confidence interval (bars); HOMA2-%B, homeostasis model assessment 2-beta cell function

Figure S15. Low carbohydrate versus low fat diet and ALT

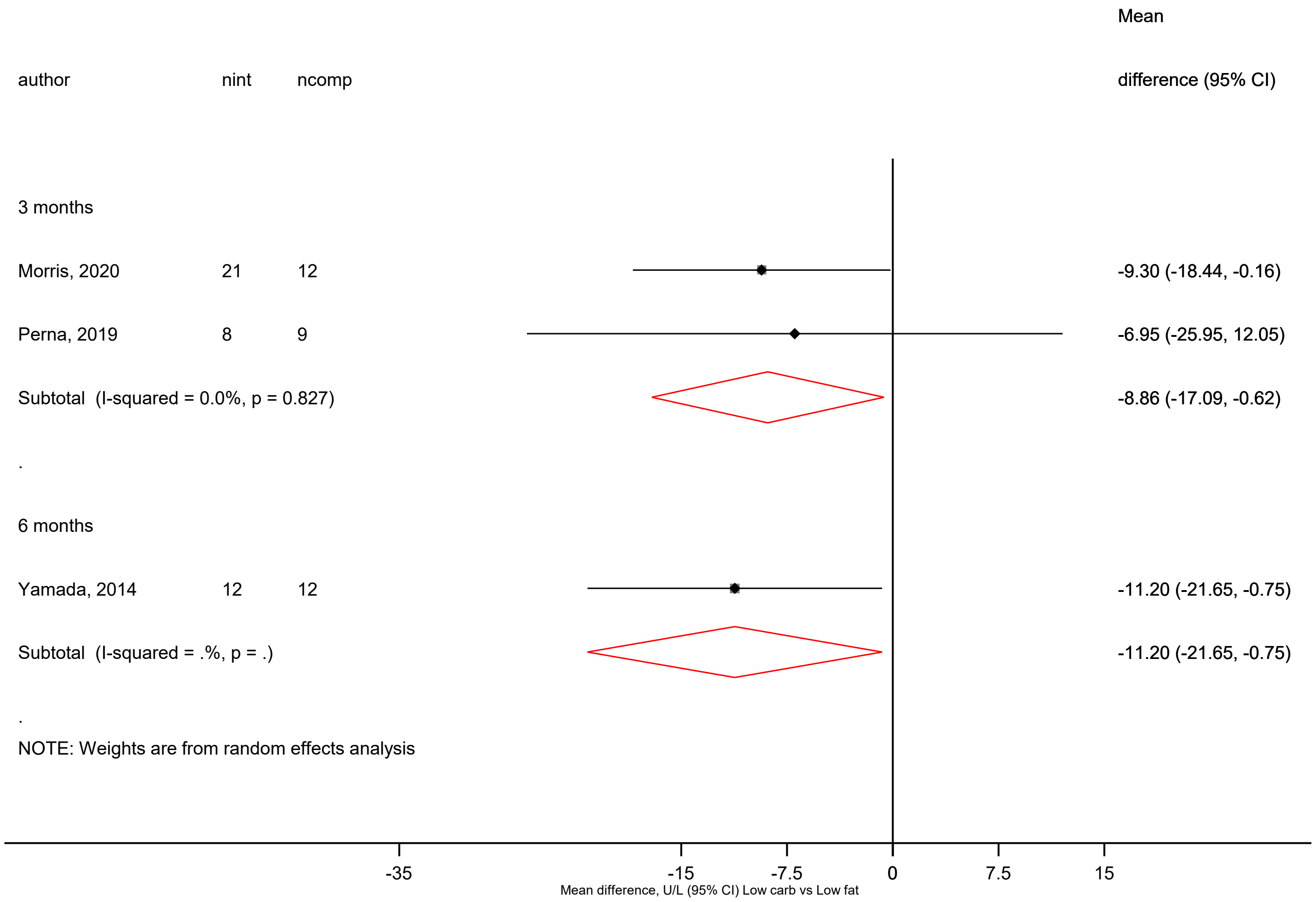

ALT, alanine aminotransferase; CI, confidence interval

Figure S16. Low carbohydrate versus low fat diet and AST

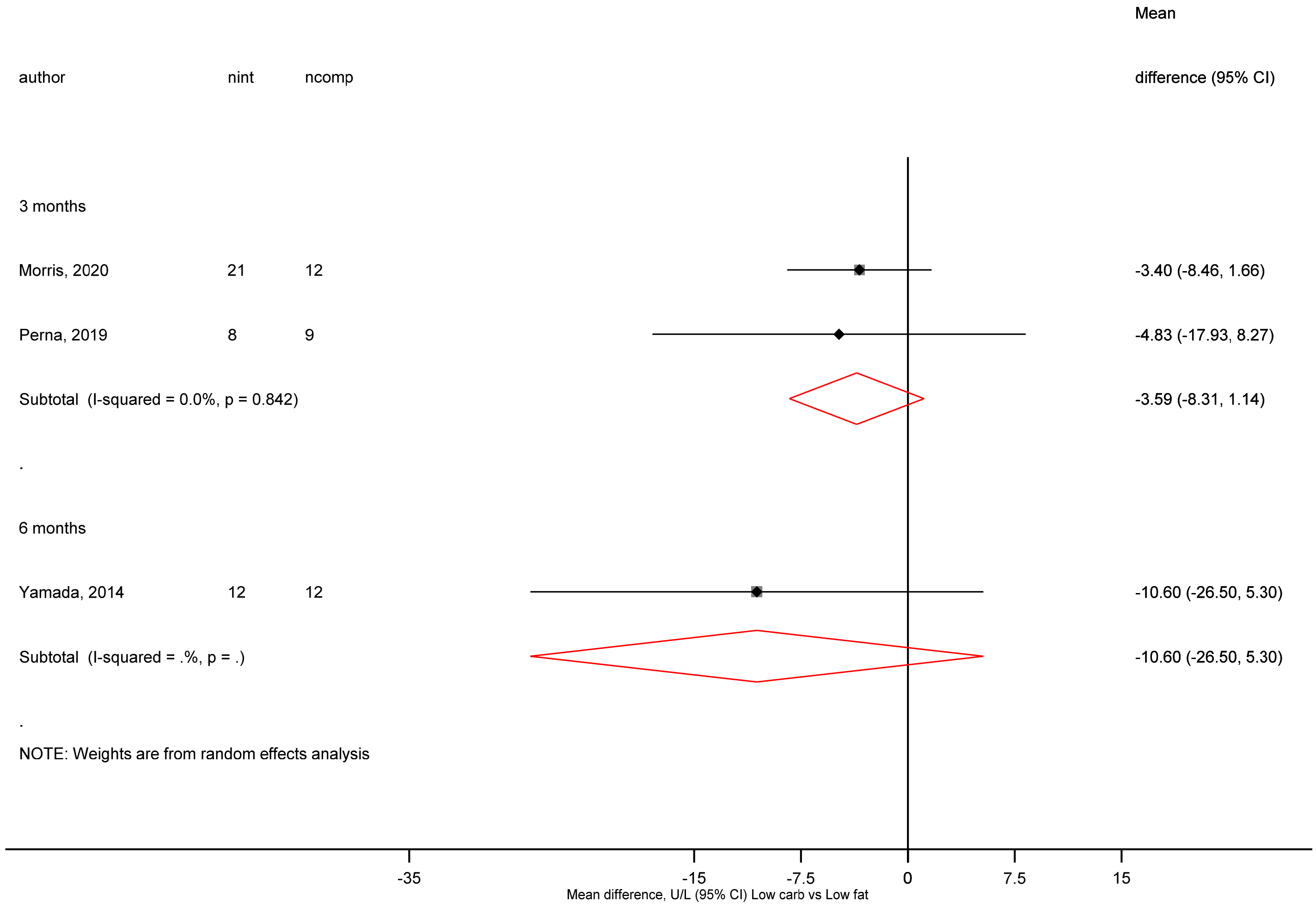

AST, aspartate aminotransferase; CI, confidence interval

Figure S17. Low carbohydrate versus low fat diet and GGT

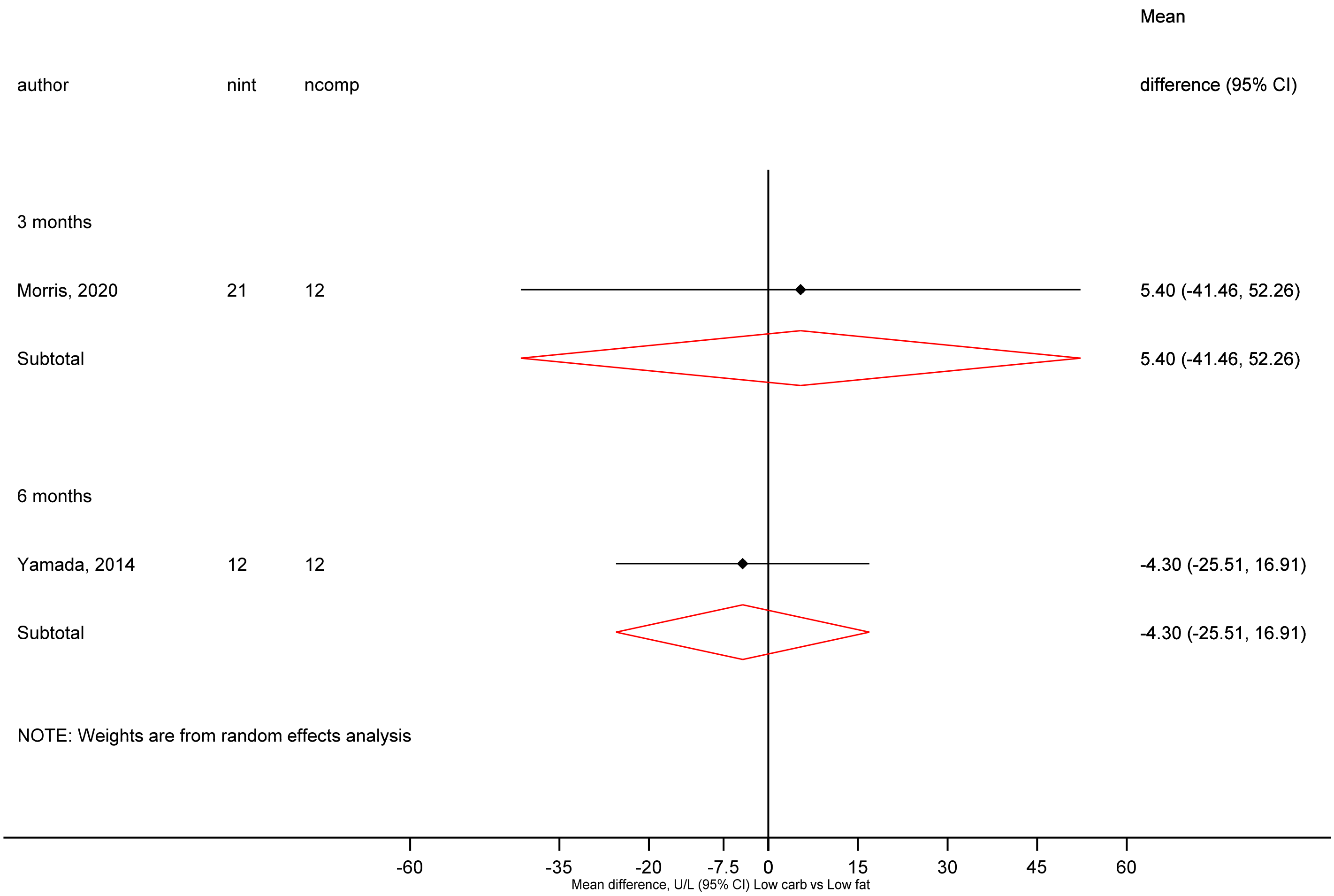

CI, confidence interval; GGT, gamma glutamyltransferase

Figure S18. Low carbohydrate versus low fat diet and creatinine

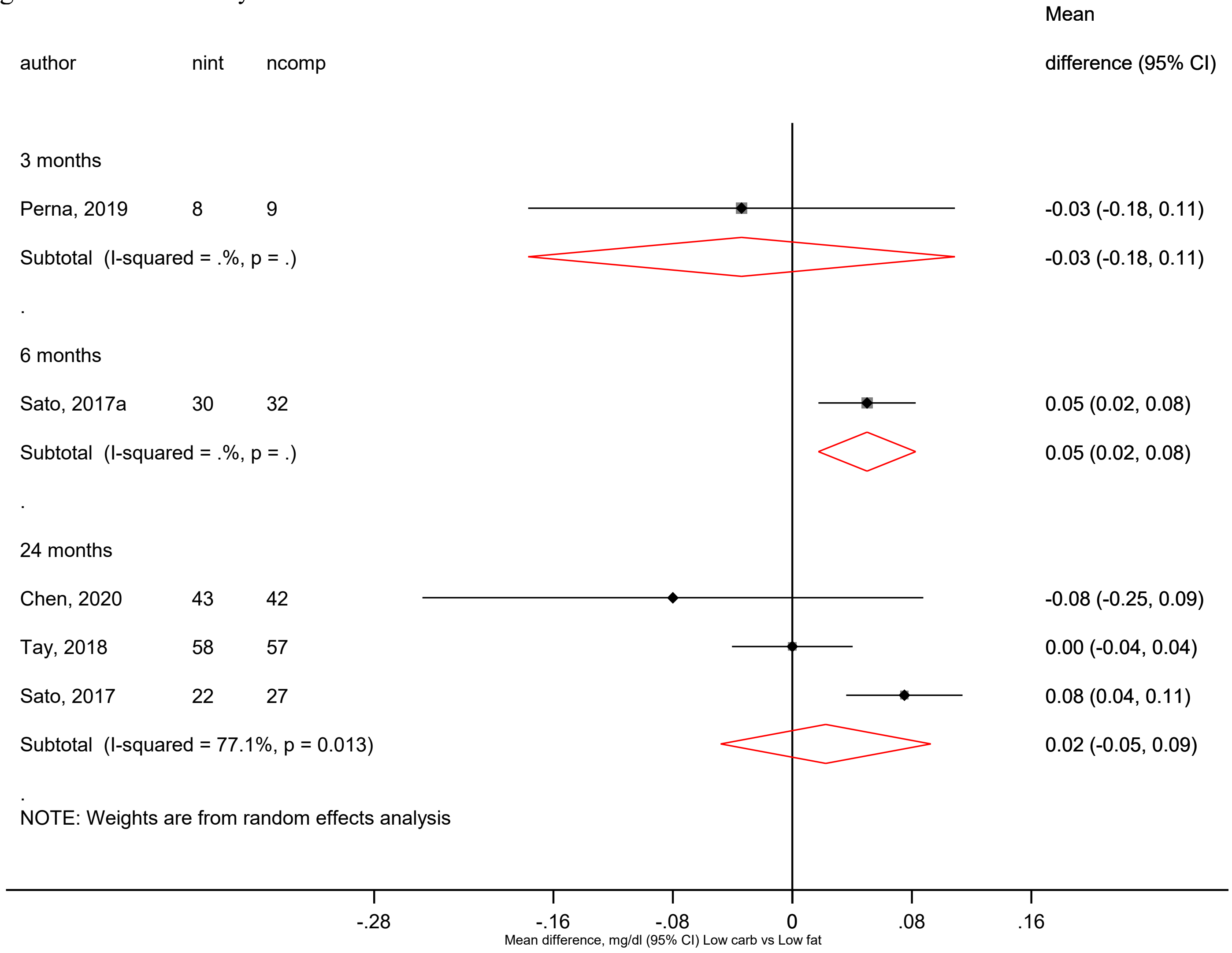

CI, confidence interval

Figure S19. Low carbohydrate versus low fat diet and estimated GFR

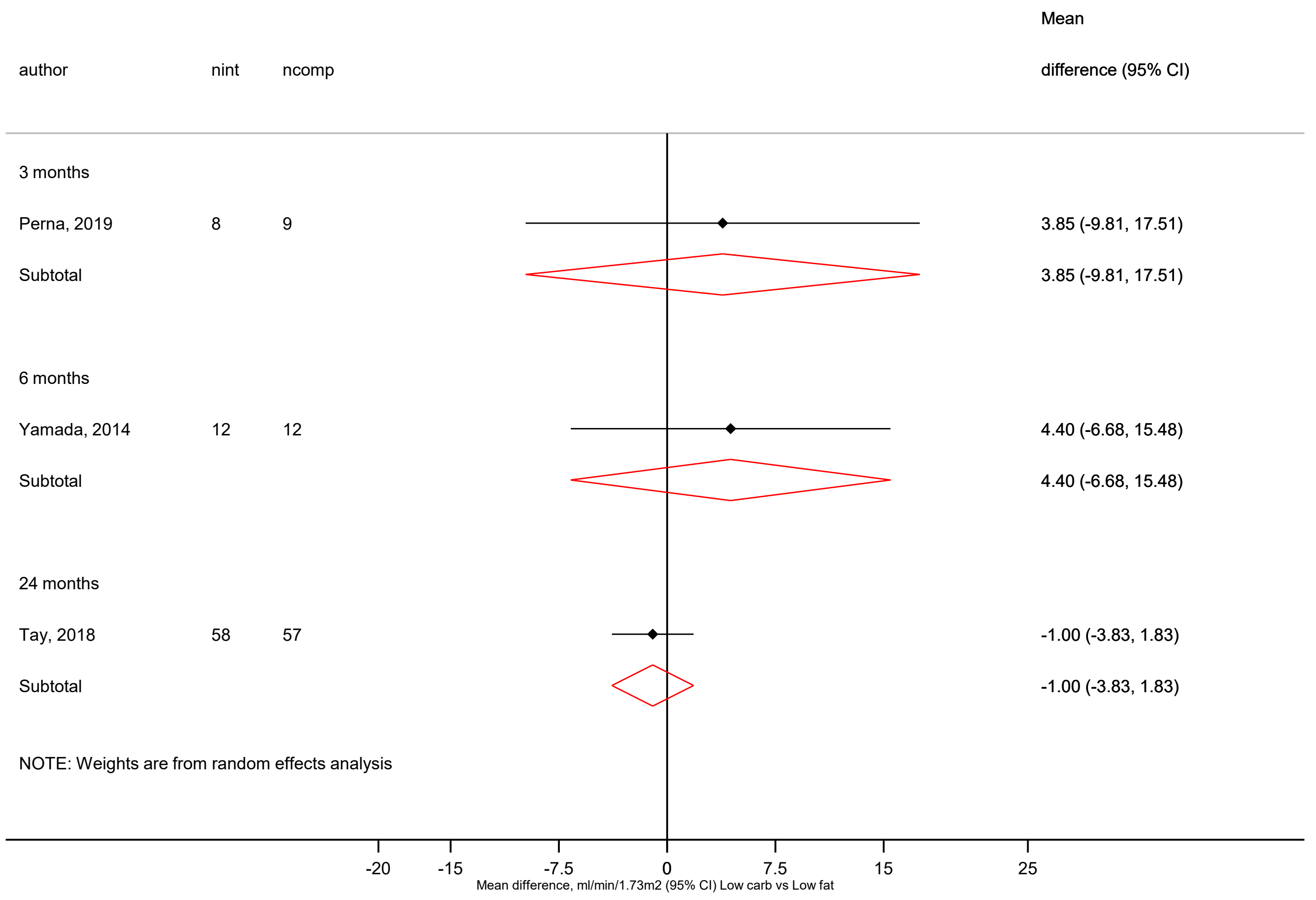

CI, confidence interval; GFR, glomerular filtration rate

Figure S20. Low carbohydrate versus low fat diet and microalbumin

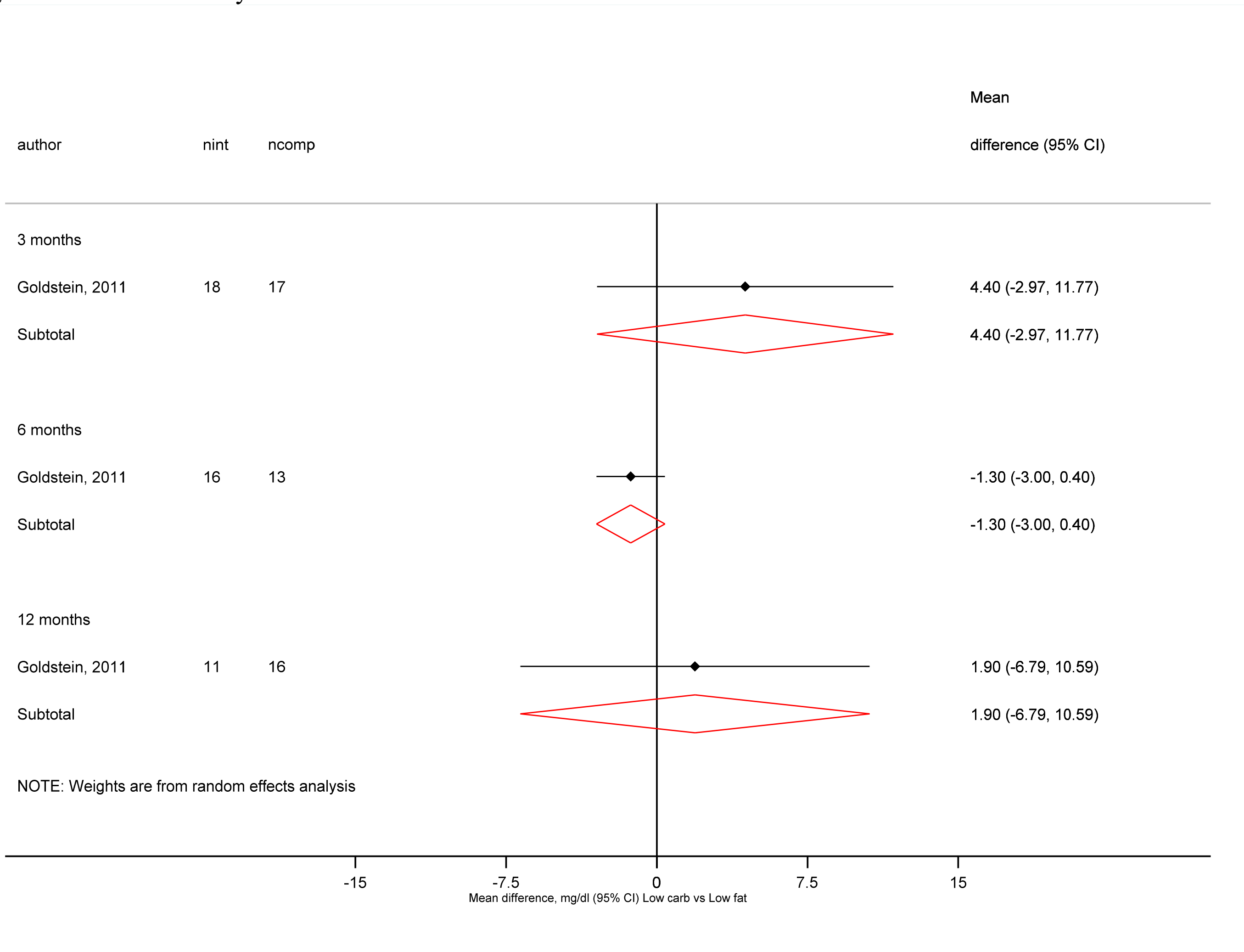

CI, confidence interval

Figure S21. Low carbohydrate versus low fat diet and urea

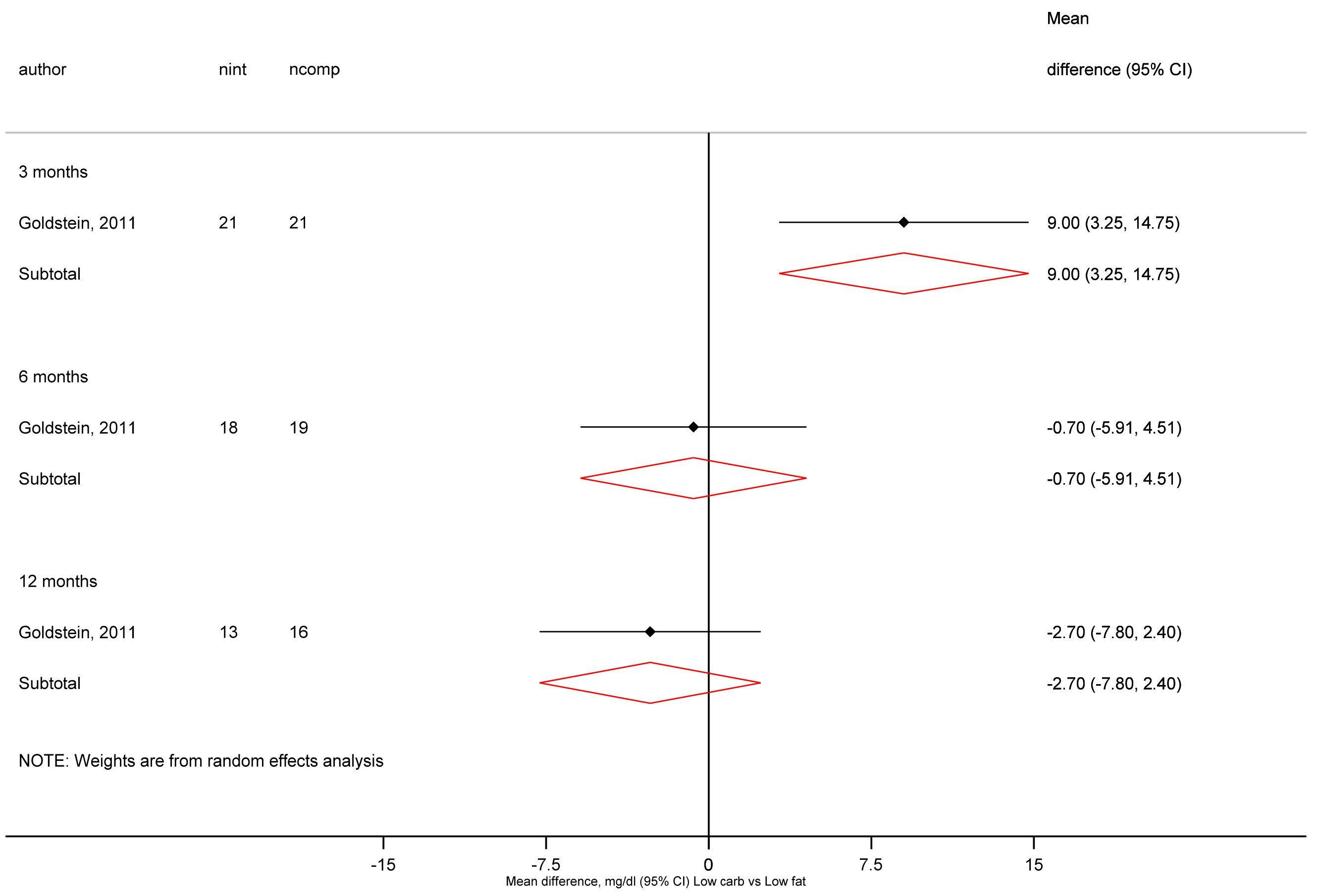

CI, confidence interval

Figure S22. Low carbohydrate versus low fat diet and urinary albumin

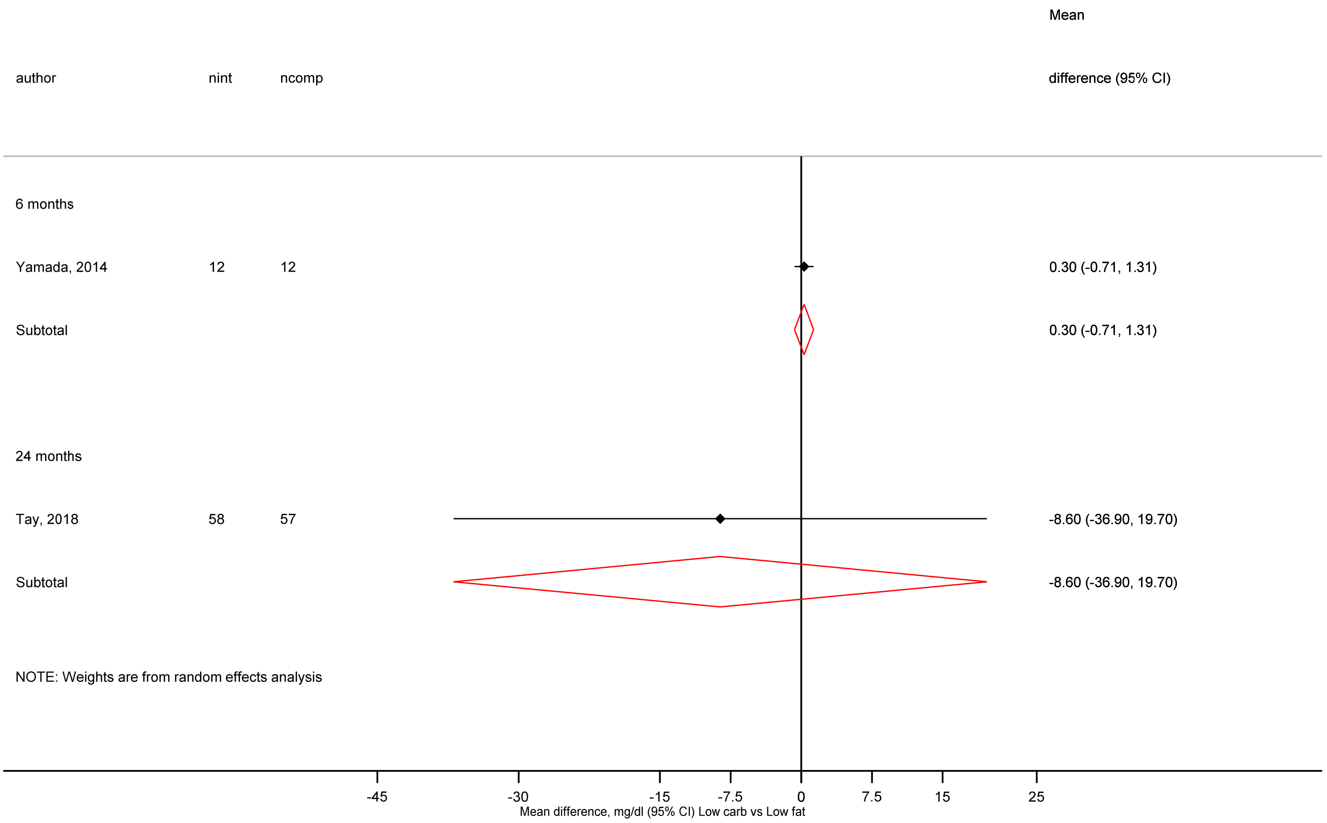

CI, confidence interval

Figure S23. Low carbohydrate versus low fat diet and uric acid

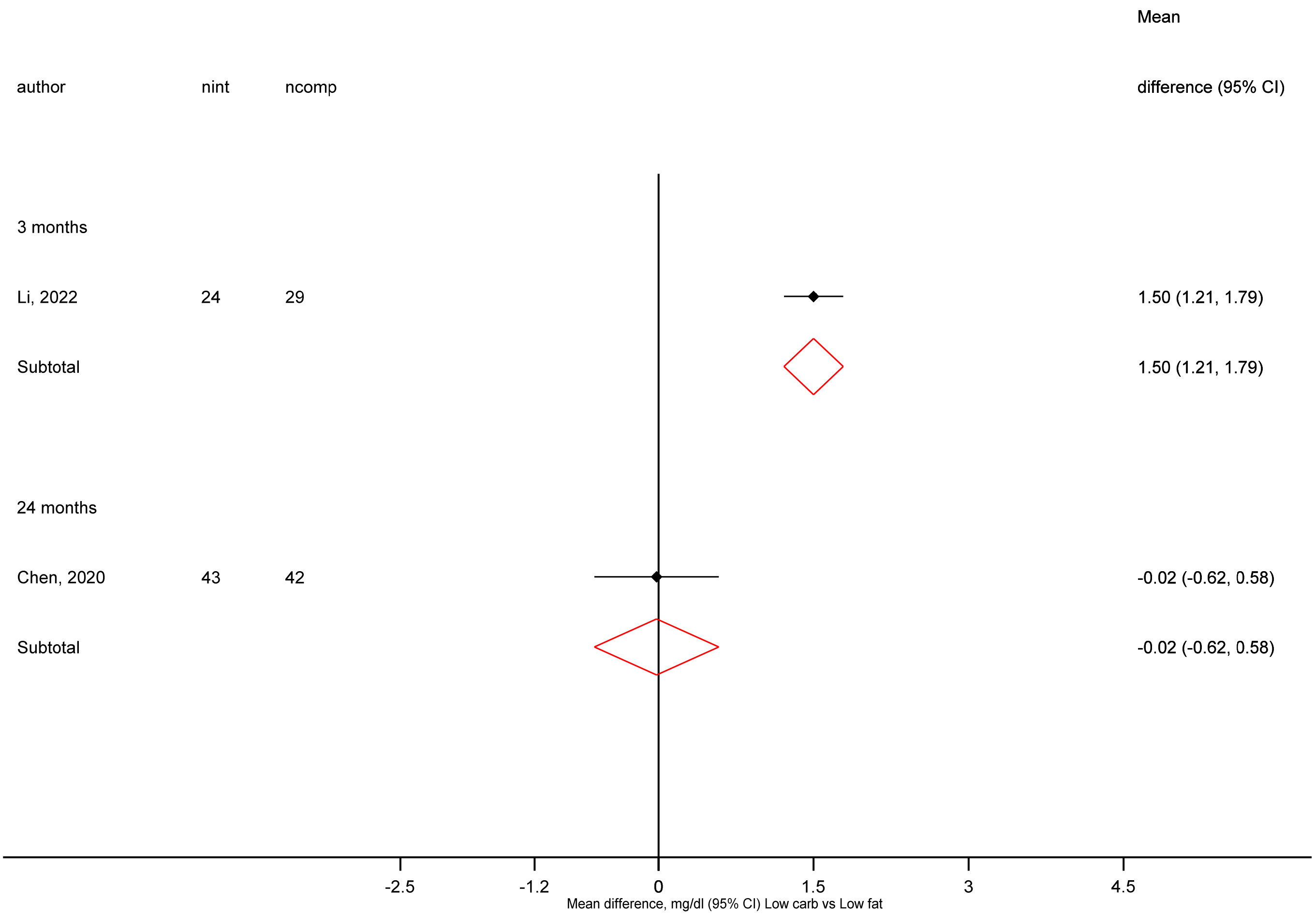

Figure S24. Low carbohydrate versus low fat diet and medication effect score

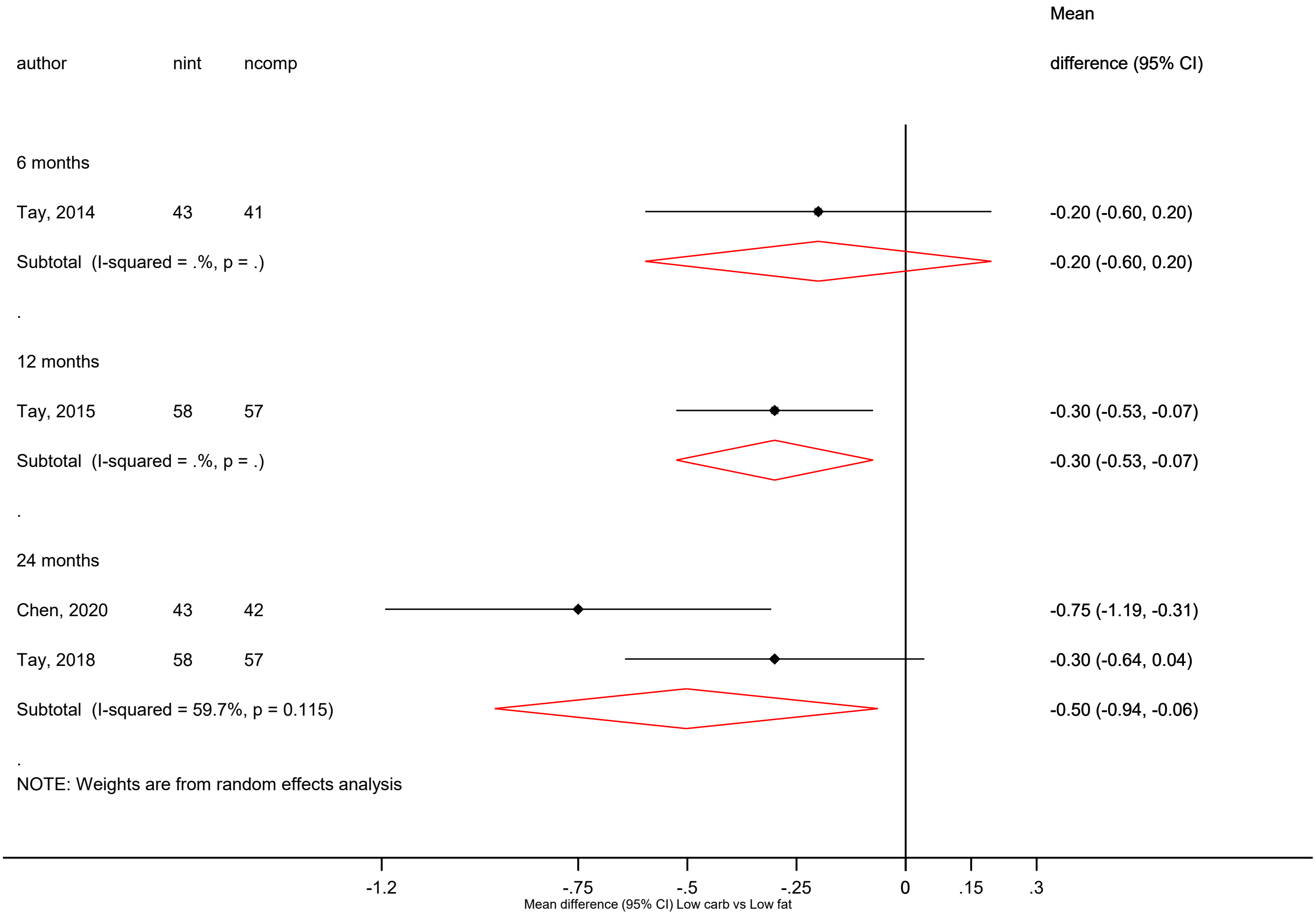

CI, confidence interval

Figure S25. Low carbohydrate versus low fat diet and risk of adverse events

author	nint	eventint	ncomp	eventcomp	RR (95% CI)	% Weight
Tay, 2014; Tay 2015	58	8	57	13	0.60 (0.27, 1.35)	23.73
Westman, 2008	105	45	145	38	1.64 (1.15, 2.32)	41.57
Yamada, 2014	12	3	12	0	7.00 (0.40, 122.44)	3.31
Tay, 2018	58	11	57	10	1.08 (0.50, 2.35)	24.72
Gram-Kampmann, 2021	49	12	22	1	2.94 (0.42, 20.45)	6.67
Overall (I-squared = 44.8%, p = 0.123)					1.27 (0.74, 2.18)	100.00

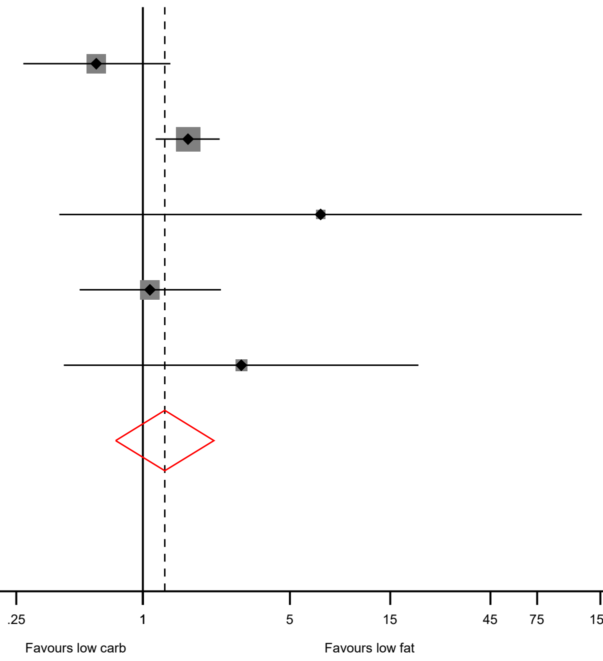

CI, confidence interval; RR, relative risk

Table S4. GRADE summary of findings

Outcomes	№ of participants (studies) Follow-up	Certainty of the evidence (GRADE)	Relative effect (95% CI)	Anticipated absolute effects	
				Risk with [Low fat diet]	Risk difference with [Low carb diet]
Fasting glucose	335 (5 RCTs)	⊕◯◯◯ Very low[a,b,c]	-		
HbA1c	582 (9 RCTs)	⊕◯◯◯ Very low[a,d]	-		
Body weight	586 (12 RCTs)	⊕⊕⊕◯ Moderate[a]	-		
Body mass index	413 (7 RCTs)	⊕⊕⊕◯ Moderate[a]	-		
Systolic blood pressure	581 (8 RCTs)	⊕◯◯◯ Very low[a,c,d]	-		
Total cholesterol	553 (7 RCTs)	⊕⊕⊕◯ Moderate[a]	-		
Adverse events	586 (5 RCTs)	⊕⊕◯◯ Low[a,c]	**RR 1.27** (0.74 to 2.18)	270 per 1,000	**73 more per 1,000** (70 fewer to 318 more)

***The risk in the intervention group** (and its 95% confidence interval) is based on the assumed risk in the comparison group and the **relative effect** of the intervention (and its 95% CI).
CI: confidence interval; **RR:** risk ratio

GRADE Working Group grades of evidence
High certainty: we are very confident that the true effect lies close to that of the estimate of the effect.
Moderate certainty: we are moderately confident in the effect estimate: the true effect is likely to be close to the estimate of the effect, but there is a possibility that it is substantially different.
Low certainty: our confidence in the effect estimate is limited: the true effect may be substantially different from the estimate of the effect.
Very low certainty: we have very little confidence in the effect estimate: the true effect is likely to be substantially different from the estimate of effect.

Explanations

a. High risk of bias in at least 2 domains

b. I-squared value of 73%

c. Wide confidence intervals

d. I-squared value>90%
